# Supplementary material for: Disruption of tRNA threonylation triggers RIG-I mediated anti-tumour immune response
Source: Nat Commun. 2026 Feb 25;17:3145. doi: 10.1038/s41467-026-69964-2 (PMC13043769; doi:10.1038/s41467-026-69964-2)

Supplementary Information

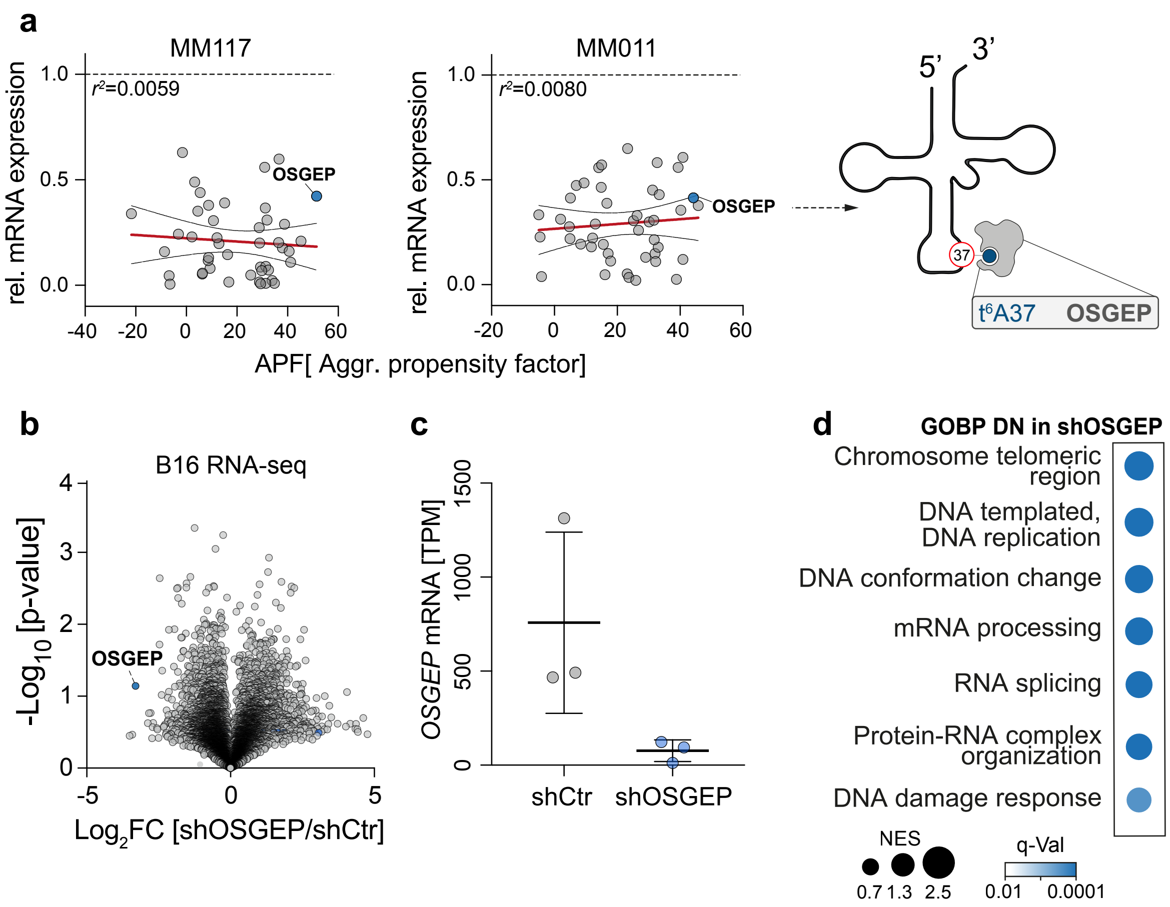

Supplementary Figure 1: Loss of OSGEP compromises proteostasis and delays tumor growth in melanoma.

a. Correlation plot between mRNA expression of 4 tRNA modification enzymes after esiRNA depletion and protein aggregation level (APF) in MM117 and MM011 human melanoma cell line. The data show no correlation between the levels of enzyme depletion and APF in the two lines. **b**. Volcano plot of differential RNA expression in RNA-seq experiments in B16F10 shCTR or depleted of OSGEP (shOSGEP). **c**. Quantification of OSGEP expression in shCtr or shOSGEP B16F10 from the RNA-seq data. **d**. Gene set enrichment analysis (GSEA) using RNA-seq data from shCtr versus shOSGEP B16F10 cells (NES: normalized enrichment score; GOBP: gene ontology biological process; down-regulated pathways (DN)). Source data are provided as a Source Data file.

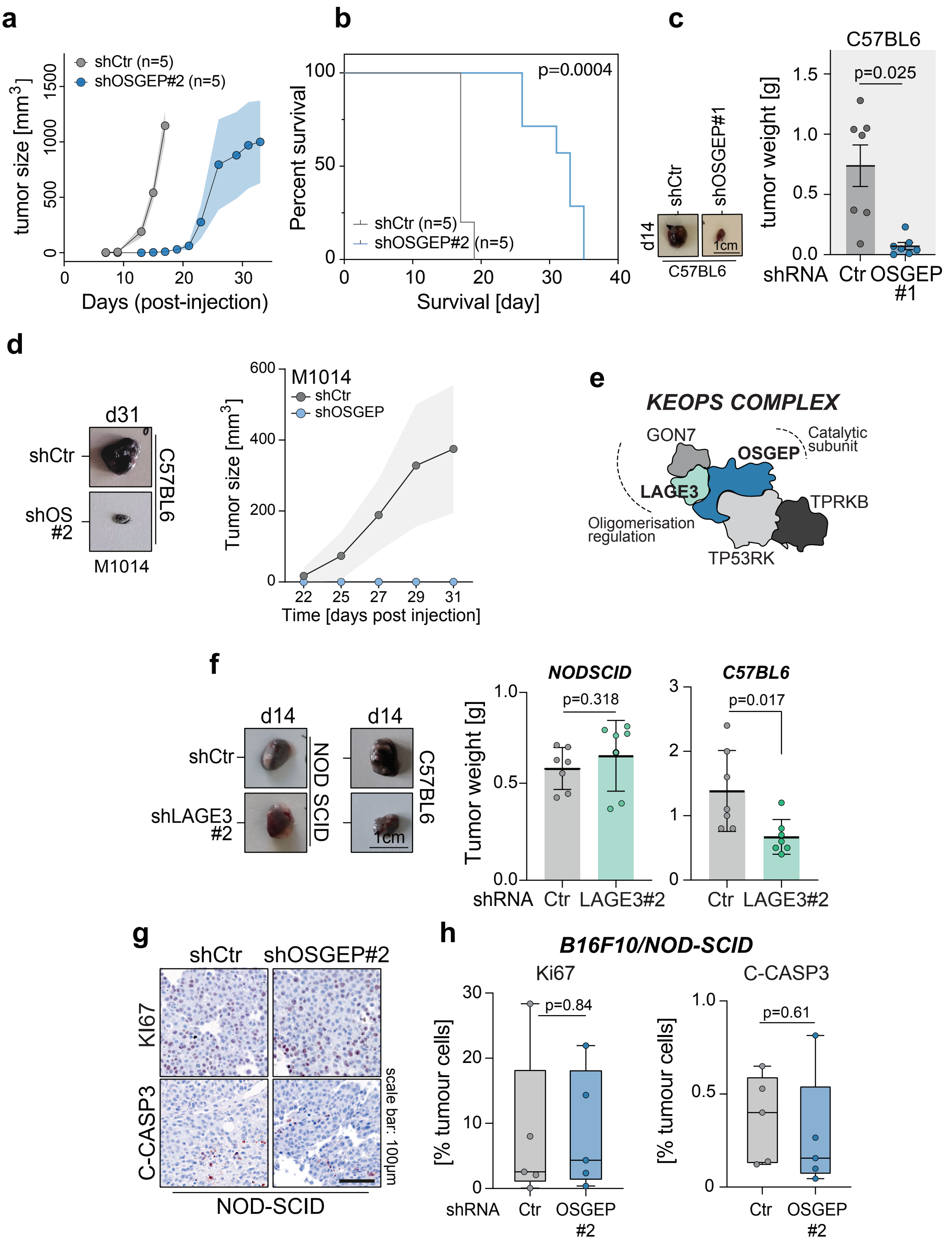

Supplementary Figure 2: KEOPS complex leads to melanoma growth defect.

a-b. Growth curve and survival curve of C57BL/6 mice bearing B16F10 tumours depleted for shCtr or shOSGEP. **c**. Representative B16F10 tumours and quantification of tumour weight 14 days after subcutaneous transplantation in C57BL/6 mice (n=7 mice per condition). Tumours derived from B16F10 cells were infected with shRNA control (shCtr) or OSGEP

(shOSGEP#1). **d**. Tumour growth of M1014 murine melanoma cell line depleted for shRNA control or OSGEP. **e**. schematic representation of KEOPS complex. **f**. Representative B16F10 tumours and quantification of tumour weight 14 days after subcutaneous transplantation in NOD SCID mice and C57BL/6 mice (n=7 mice per condition). Tumours derived from B16F10 cells were infected with shRNA control or LAGE3. **g-h**. Representative immunostainings (g) and quantification (h) of Ki67 and CASPASE-3 (CASP3) staining in B16F10-derived tumours (day 14) infected with shRNAs Ctr or targeting OSGEP (n=5 tumours per condition) from NOD SCID mice. Shown is mean +/- SD (c, f). Mantel-cox test (b), Mann Whitney test (f for nod scid; h for ki67), Unpaired two-tailed t-test (c,f,h). Exact p-values are indicated. Source data are provided as a Source Data file.

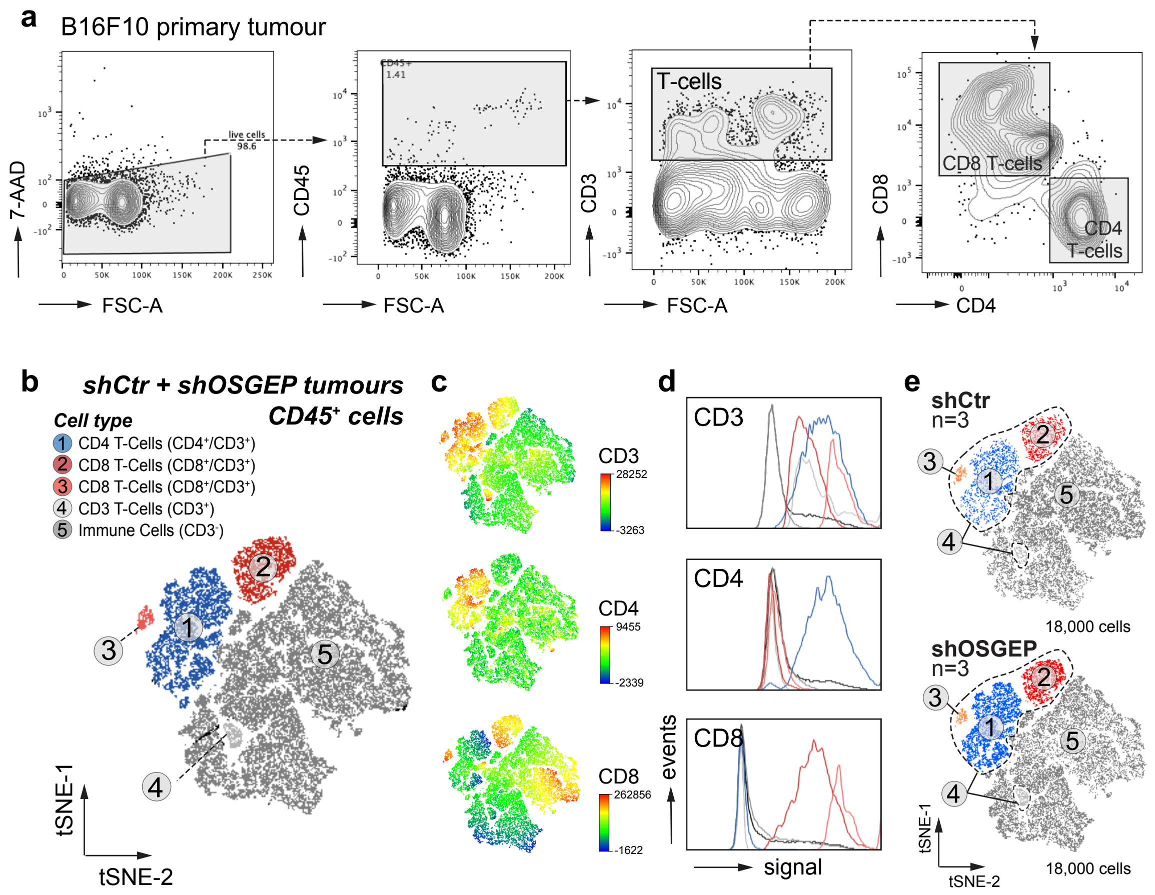

Supplementary Figure 3: OSGEP depletion leads to tumoral T-cells infiltration.

a. Gating strategy and representative plots of FACS analysis of T cell immune population from B16F10 tumours. **b-e.** t-Distributed stochastic neighbor embedding (SNE) representation of T cell population infiltration in shCtr and ShOSGEP tumours (n=3; 18,000 cells).

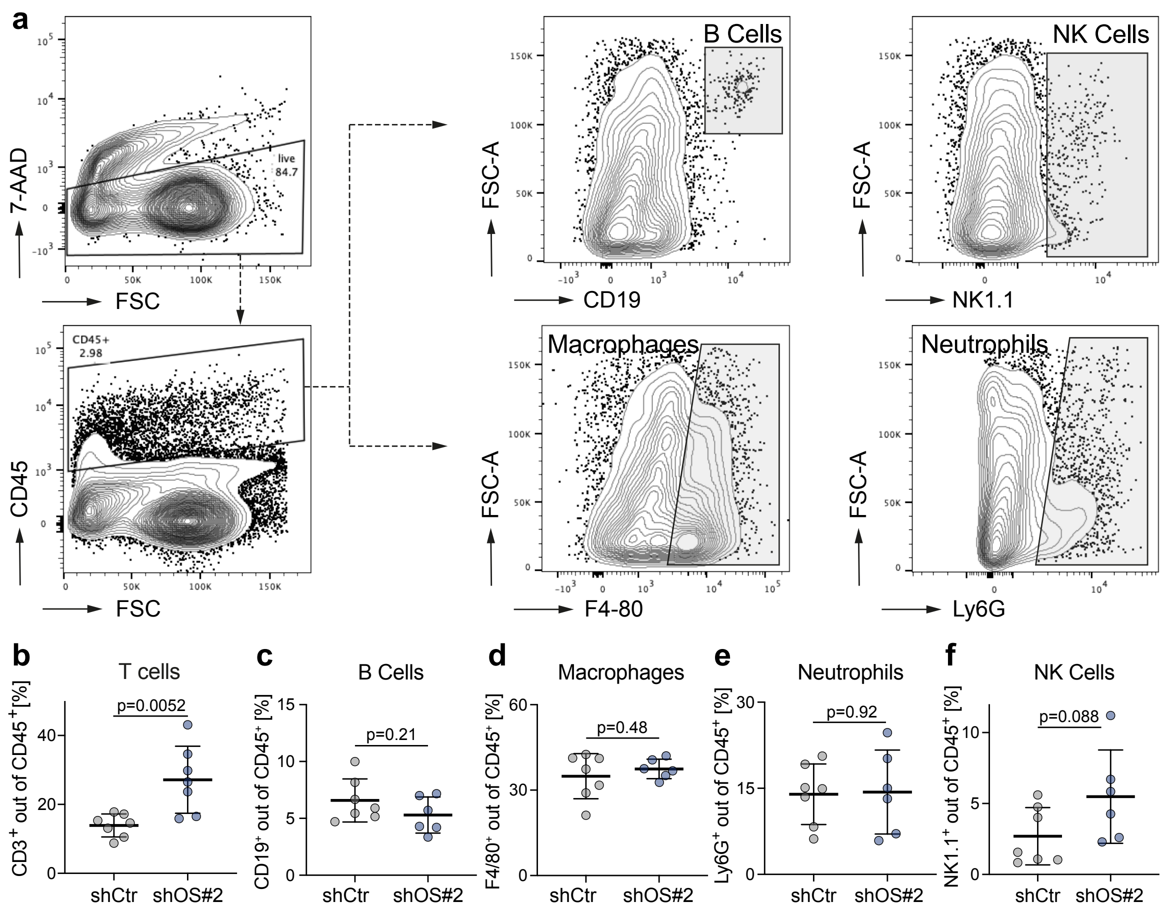

Supplementary Figure 4. Measurement of immune cells infiltration in OSGEP depleted tumor.

a. Gating strategy and representative plots of FACS analysis of indicated immune cell populations from B16F10 tumours. **b-f.** Quantification of the indicated immune cell subtypes in control (n=7) or shOSGEP#2 (n=6) depleted tumours. Shown is mean +/- SD. Unpaired two-tailed t-test. Exact p-values are indicated. Source data are provided as a Source Data file.

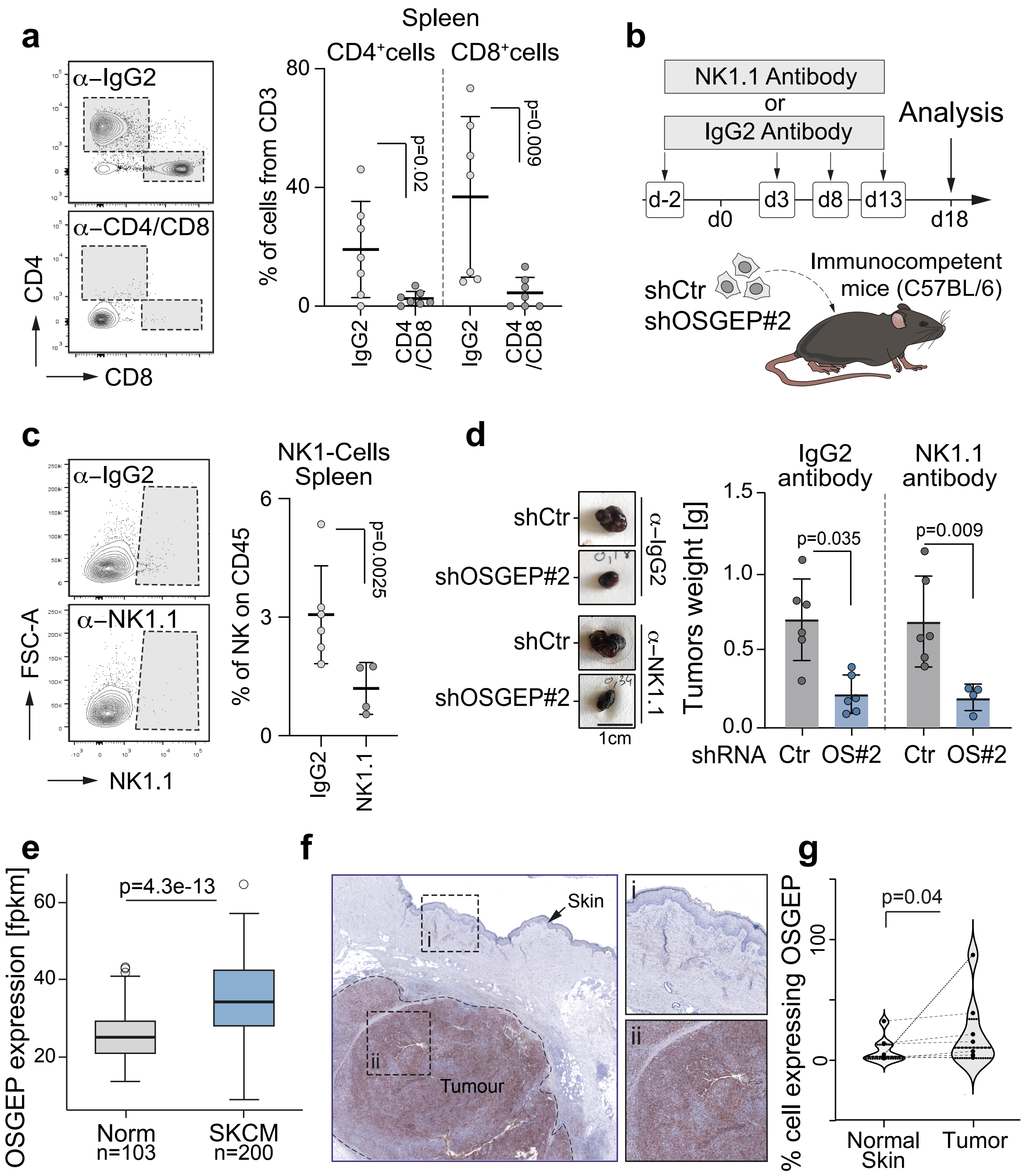

Supplementary Figure 5. In vivo depletion of immune cells.

a. FACS analysis of CD4+ and CD8+ T cell levels in spleen at end point (day14) of mice injected with IgG2 or with α-CD4/α -CD8 antibodies. **b**. Scheme of experimental procedure for the depletion of NK cells in immunocompetent mice using control (IgG2) or antibodies targeting NK cells (NK1.1), starting 2 days before subcutaneous injection of melanoma cells. **c.** Measurement of NK cells abundance by FACS analysis in the spleen at the end point mice from b. **d**. Representative B16F10 tumours and quantification of tumour weight, 1 days after subcutaneous transplantation into C57BL/6 mice treated with IgG2 (n=6 mice per condition) or NK1.1 (n=6 mice per condition) antibodies (scheme in b). Tumours derived from B16F10 cells

were infected control (shCtr) or OSGEP (shOSGEP) shRNAs. **e.** Expression of OSGEP mRNA in SKCM-TCGA (n=200) patients and normal skin tissue (n=103), extracted from the oncoDB website. **f**. Representative picture of tumours and skin delimitation in patient biopsies. OSGEP staining is in red. **g**. Violin plot of OSGEP expression in normal tissues versus tumours in patient biopsies Shown is mean +/- SD (a,c,d). Unpaired two-tailed t-test (a,c,e), Paired two-tailed t-test (d) Wilcoxon matched-pairs test (g). Exact p-values are indicated. Source data are provided as a Source Data file.

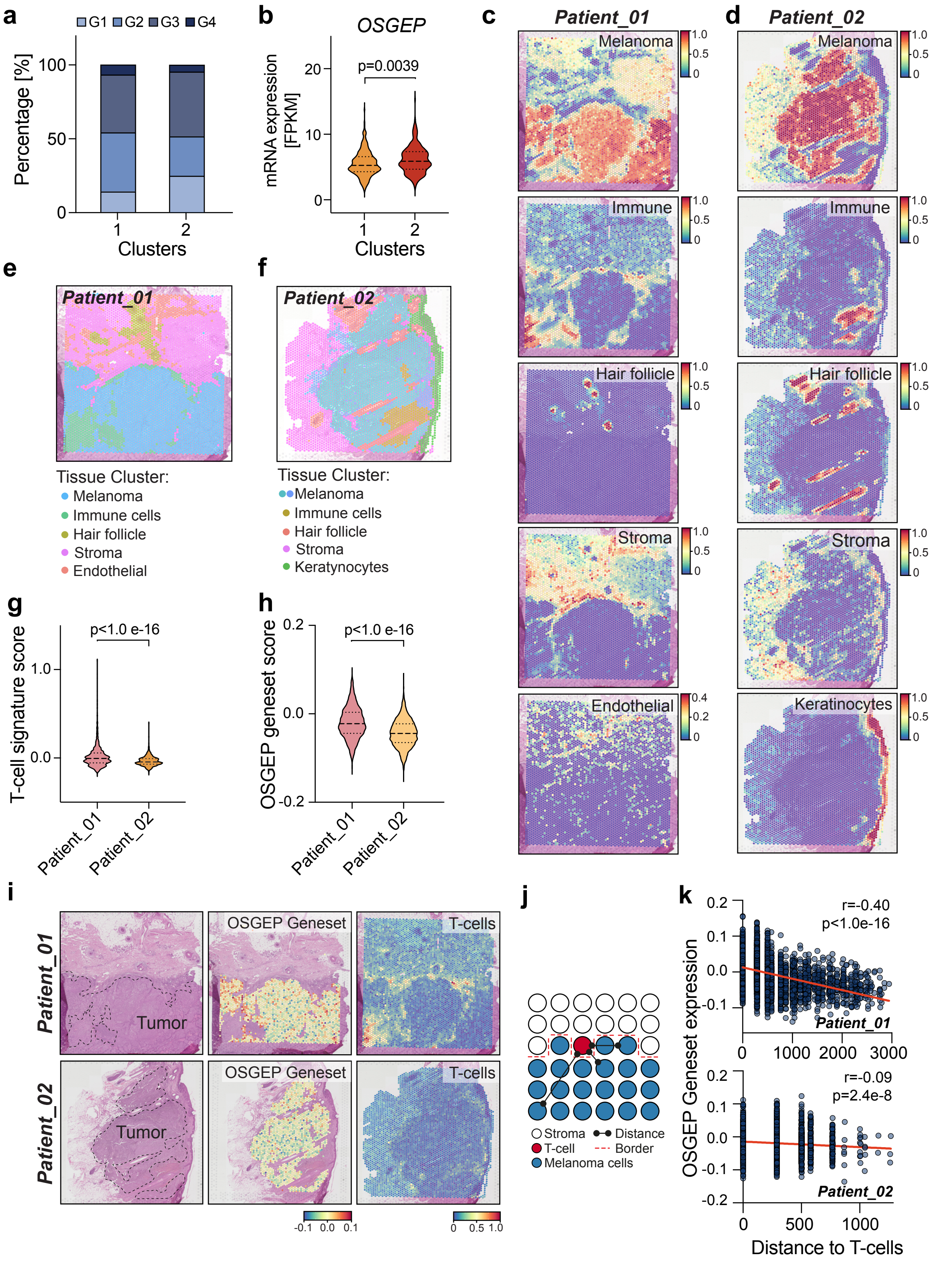

Supplementary Figure 6. OSGEP signature correlate with infiltration of T-cells in spatial transcriptomic human biopsies.

a. Frequency of the indicated pathological stages in clusters identified in (Fig. 2i) from SKCM-TCGA database. **b**. OSGEP expression in TCGA patient cluster by low or high OSGEP signature expression. **c**. Deconvolution of spatial transcriptomic on Patient_01 showing

melanoma signature, immune signature, hair follicle, stroma and endothelial cells. **d**. Deconvolution spatial transcriptomic on Patient_02 showing melanoma signature, immune signature, hair follicle, stroma and endothelial cells. **e**. Merged signatures of melanoma, immune, hair, stroma and endothelial marker clustering region in Patient_01. **f**. Merged signatures of melanoma, immune, hair, stroma and endothelial marker clustering region in Patient_02. **g**. Violin plot showing T cell signature score of Patient_01 and Patient _02. **h**. Violin plot representing OSGEP signature expression in Patient_01 and Patient 2. **i**. Representation of OSGEP signature expression and T cell infiltration in tumours of Patient_01 and _02. **j**. Schematic representation of T cell distance calculation. **k**. Correlation between OSGEP signature expression and T cell distance in Patient_001 and Patient_02. Source data are provided as a Source Data file.

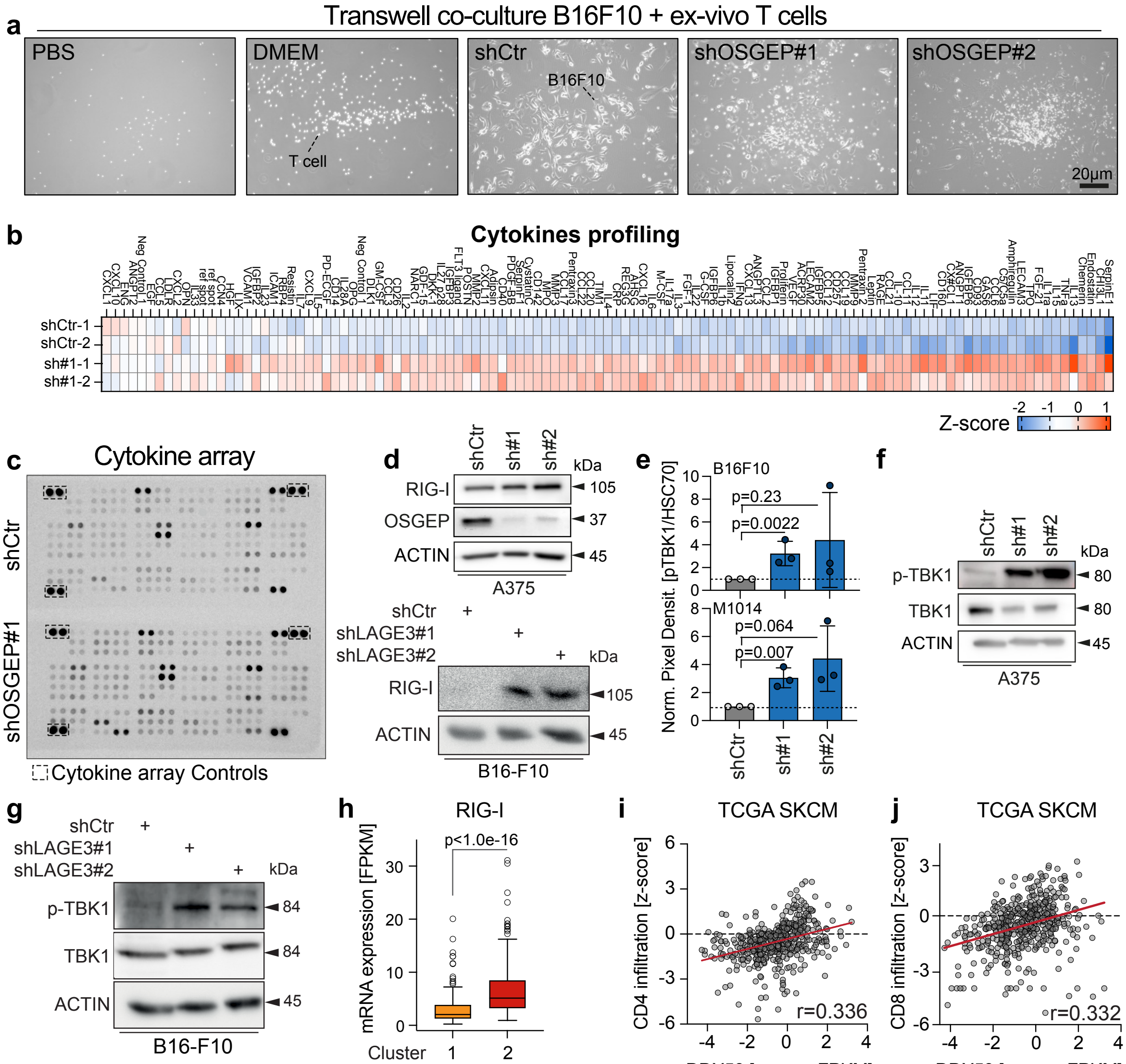

Supplementary Figure 7. OSGEP loss leads to increased cytokine production.

a. Representative images of CD3 T cell passed through Transwell© (1h) in lower chamber with or without B16F10, transduced with shRNACtrl or shOSGEP (#1,#2). **b**. Heat map of 111 cytokines detected by the cytokine array. Each square represents a pixel intensity of the dot. **c**. Membranes of the cytokine array. Each dots represent a duplicate cytokine. Three housekeeping controls were found in the upper corners and left lower corners. **d.** Densitometry calculated for p-TBK1 on housekeeping gene, in M1014 and B16F10 cells depleted for OSGEP. **e**. Western blot of RIG-I, p-TBK1 and TBK1 in human A375 melanoma cells depleted with shRNA control or OSGEP. **f**. Western blot of RIG-I in B16F10 cell depleted with shRNA control or LAGE3. **g**. Western blot of p-TBK1 and TBK1 in B16F10 cell depleted with shRNA control or LAGE3. **h**. RIG-I mRNA expression in cluster 1 and 2 (cfr Fig. 2i). **i-j.** Correlation plot between RIG-I mRNA expression and $CD4^+$ (i) or $CD8^+$ (j) infiltration score in TCGA_SKCM patient database. Data are reported as mean +/- SD (d,h). Unpaired two-tailed t-test (d,h). Exact p-value is indicated. Source data are provided as a Source Data file.

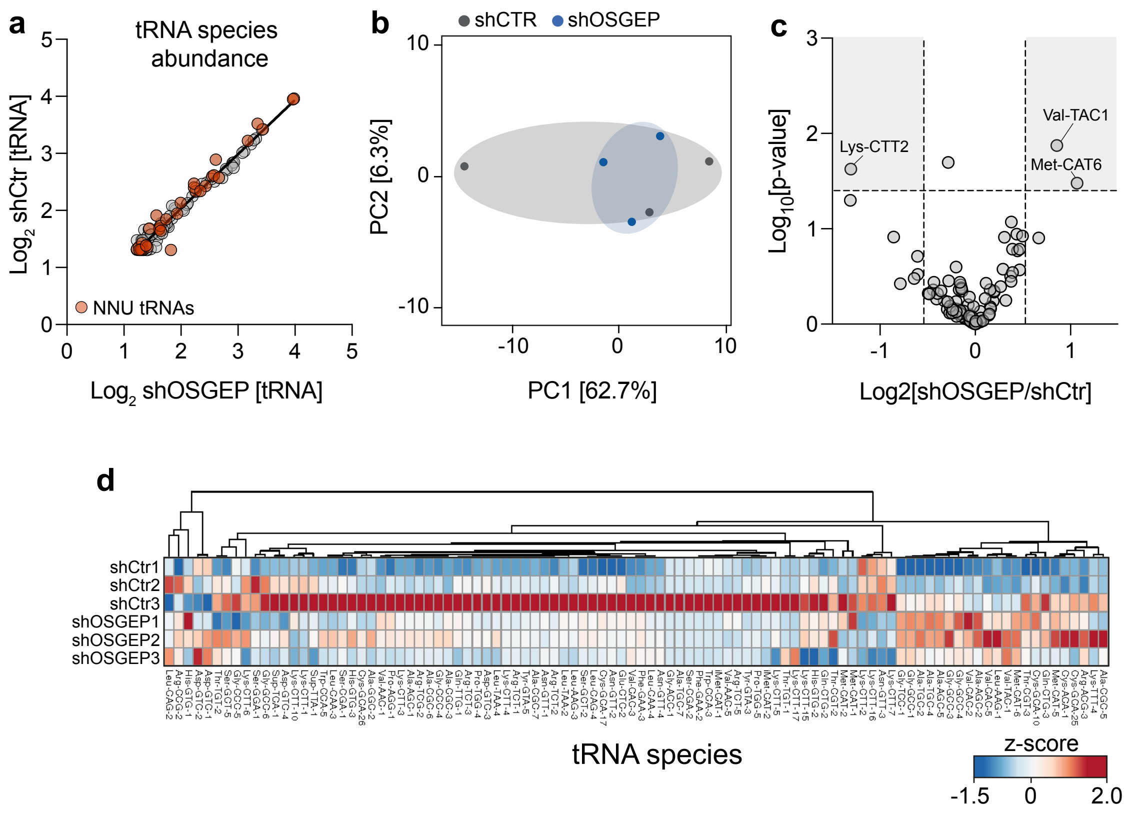

Supplementary Figure 8. Loss of t^6A does not affect tRNA pool.

a. Linear regression representation of tRNA sequencing on B16F10 cells depleted for Ctrl or OSGEP. **b**. Principal Component Analysis ((PCA) plot showing similarities between shCtr and shOSGEP B16F10 analyzed by tRNA sequencing. **c**. Volcano plot representing tRNA

sequencing from OSGEP or control depleted B16F10. Log2 fold change of OSGEP vs ctrl is shown. **d**. Heatmap representing each tRNA sequenced in each replicate of shCtr or shOSGEP B16F10. Source data are provided as a Source Data file.

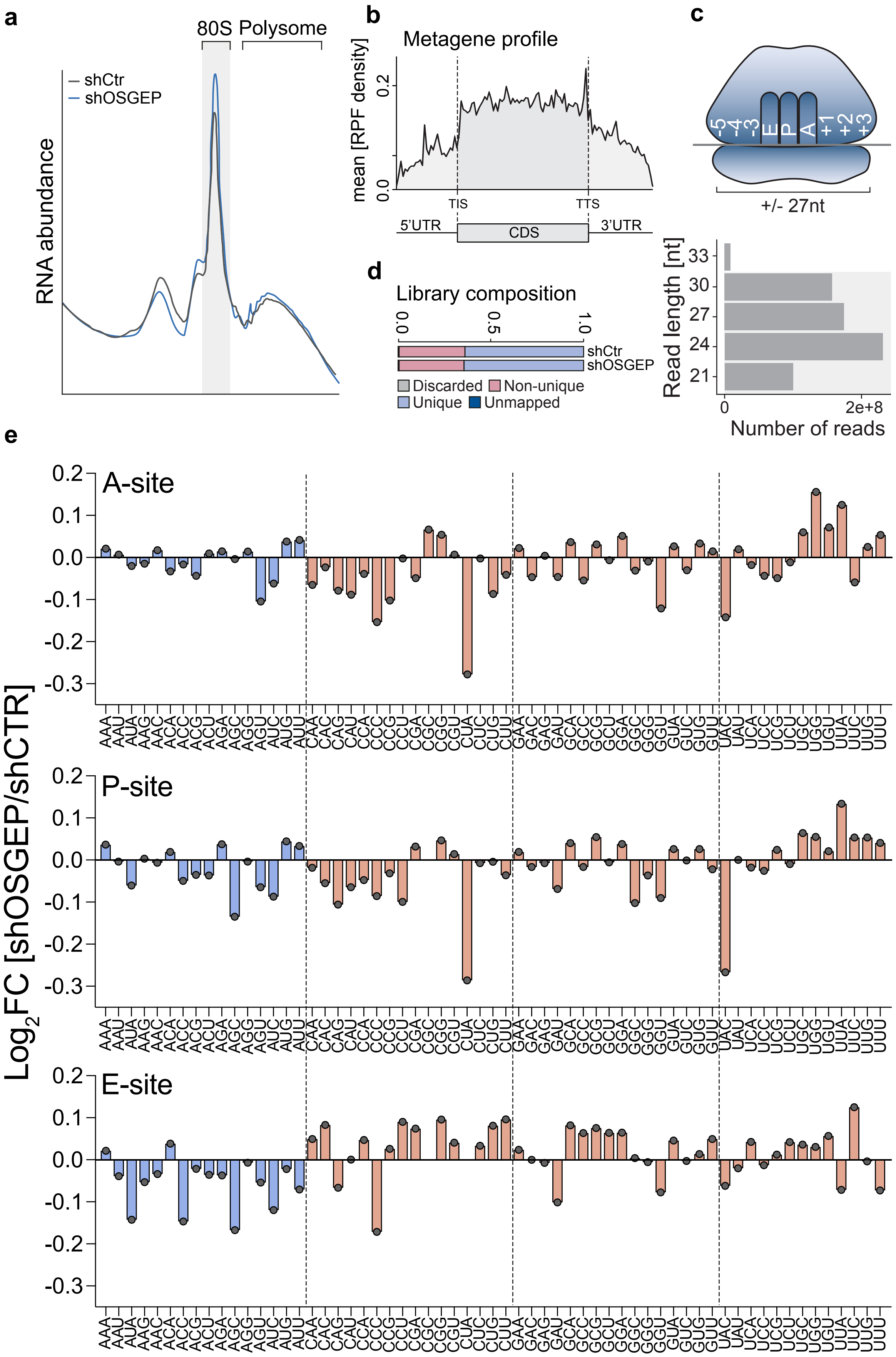

Supplementary Figure 9. Loss of t^{6}A does not affect global protein synthesis

a. Polysome profiling experiments of control or OSGEP depleted B16F10. **b**. Metagene plot providing profiles of the ribosome density average on transcripts. **c**. Schematic representation of mRNA transcript covered by a ribosome. **d**. Library composition and average read length of transcript analyzed by RNA sequencing. **e**. Bar plot representing the $\log_2$ Fold Change of the Ribosome Foot Print (RPFs) density at each codons on A, P, E sites in B16F10 shCtrl and shOSGEP. Source data are provided as a Source Data file.

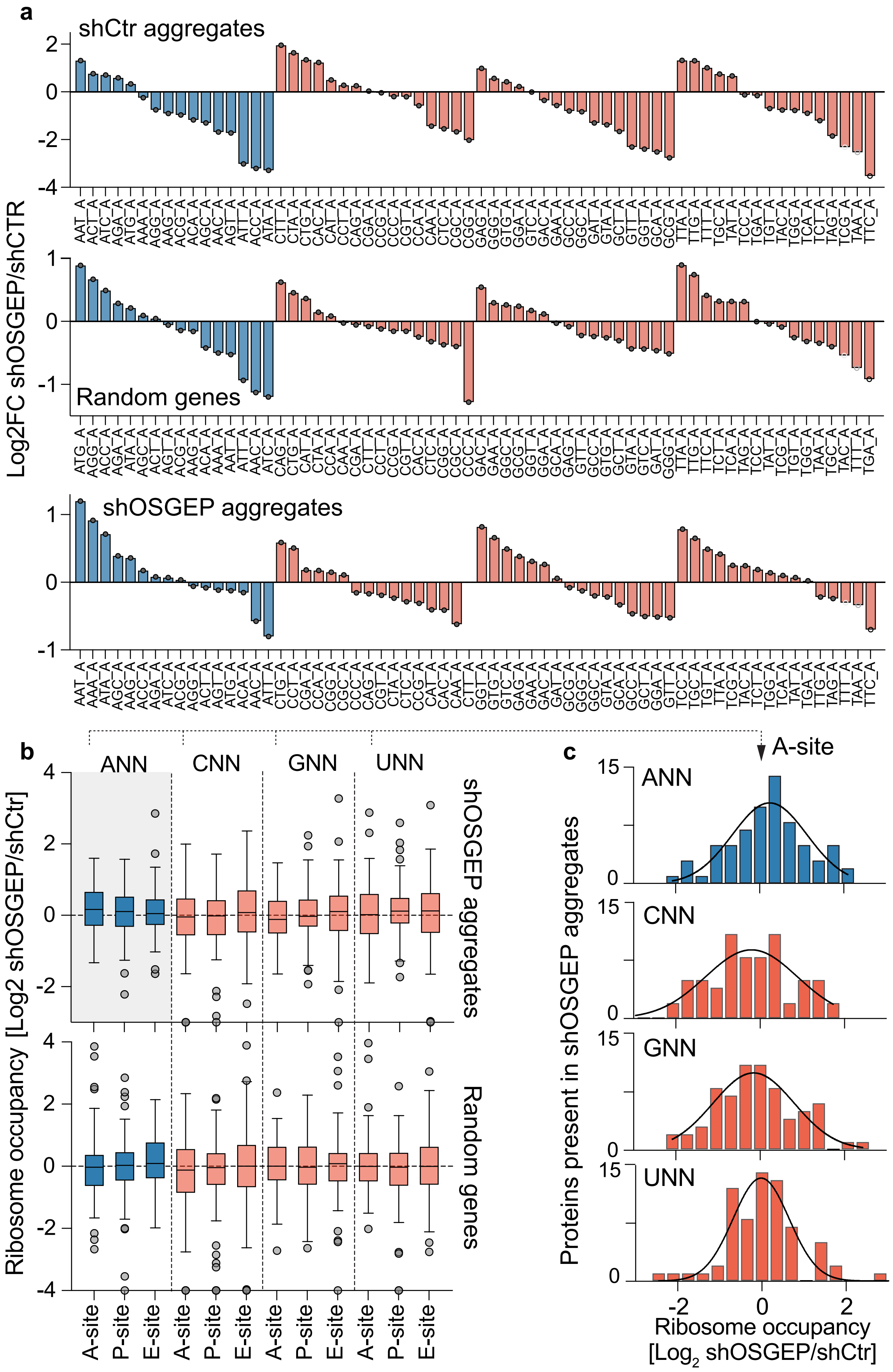

a
shCtr aggregates
Random genes
shOSGEP aggregates
Log2FC shOSGEP/shCTR
b
ANN
CNN
GNN
UNN
shOSGEP aggregates
Random genes
Ribosome occupancy [Log2 shOSGEP/shCtr]
A-site
P-site
E-site
c
A-site
Proteins present in shOSGEP aggregates
Ribosome occupancy
[Log2 shOSGEP/shCtr]

Supplementary figure 10: ANN decoding is affected in OSGEP protein aggregates

a. Bar plot of Ribosome occupancy for each codon in aggregates from control, OSGEP depleted cells or random genes. **b**.Box plot representing the log_2Fold Change of RPFs density at all codons starting with A, C, G or T (ANN, CNN, GNN, TNN) in the 78 transcripts forming aggregates in OSGEP or 78 random transcripts. **b**. Bar plot analysis Log_2FC of the RPFs distribution on ANN, CNN, GNN, TNN on transcripts corresponding to OSGEP protein aggregates. Source data are provided as a Source Data file.

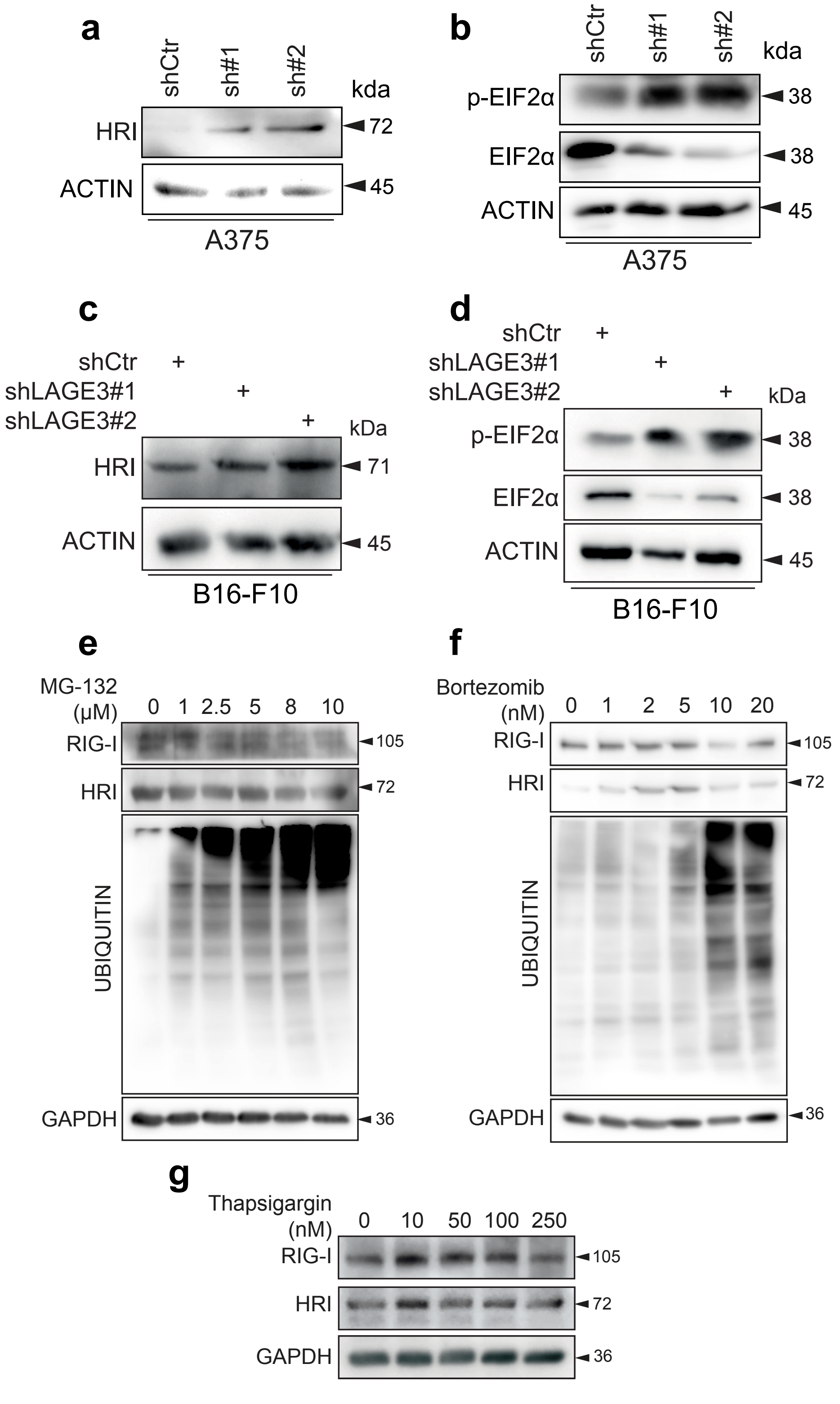

Supplementary figure 11: Loss of OSGEP in human melanoma cell lines or LAGE3 in B16F10 murine melanoma cell line recapitulate HRI activation.

a. Western blot of HRI in control or OSGEP depleted human melanoma cell line A375. **b**. Western blot of p-eIF2α and eIF2α in control or OSGEP depleted human melanoma cell line A375. **c**. Western blot of HRI in B16F10 cell depleted with shRNA control or LAGE3. **d**.

Western blot of p-eIF2α and eIF2α in B16F10 cell depleted with shRNA control or LAGE3. **e**. Western blot of RIG-I and HRI in B16F10 cells treated with indicated concentration of MG-132 for 4 hours. Ubiquitin is used to assess treatment efficacy. **f**. Western blot of RIG-I and HRI in B16F10 cells treated with indicated concentration of Bortezomib for 16 hours. Ubiquitin is used to assess treatment efficacy. **g**. Western blot of RIG-I and HRI in B16F10 cells treated with indicated concentration of Thapsigargin for 16 hours. Source data are provided as a Source Data file.

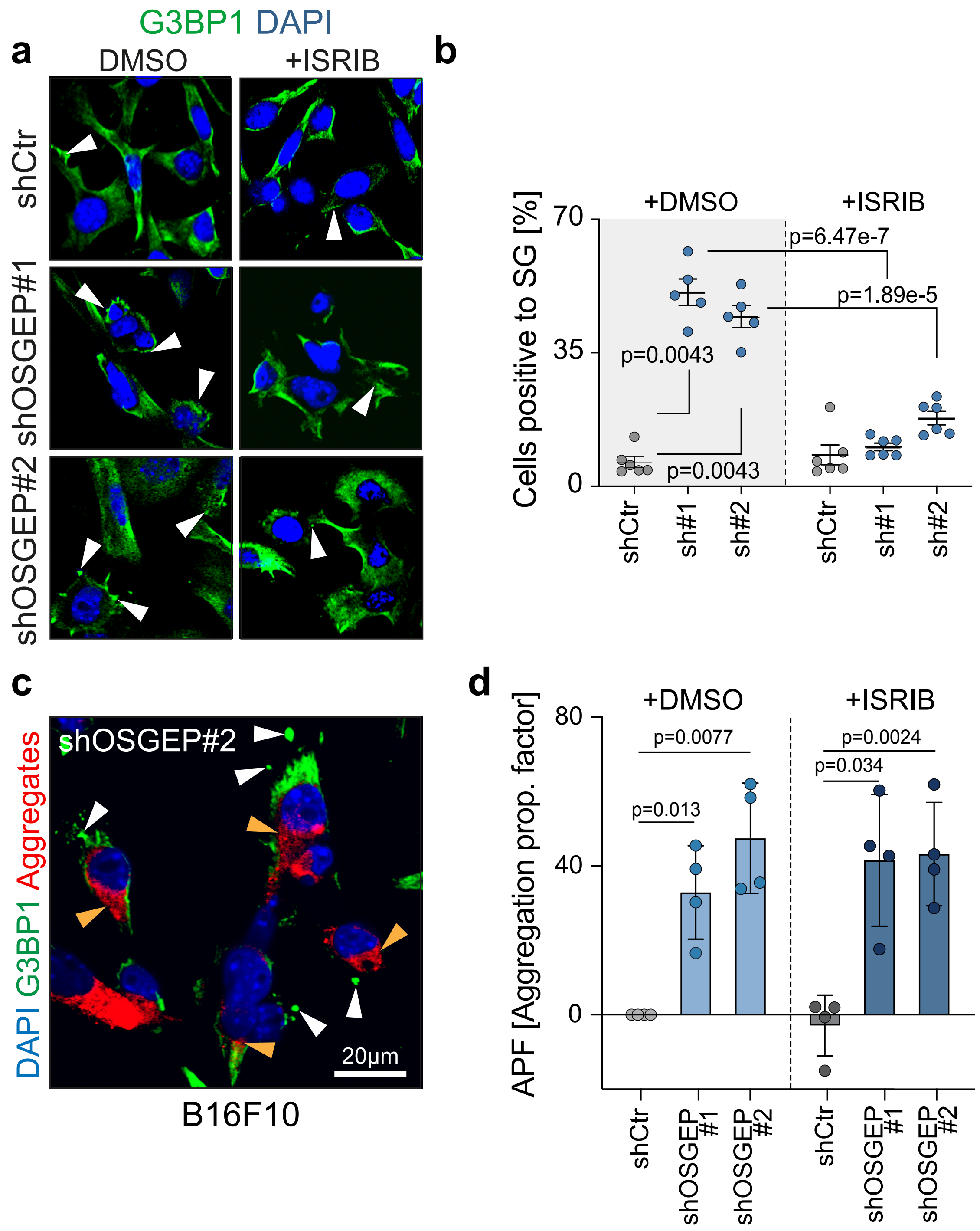

Supplementary figure 12: Protein aggregation and Stress Granules formation are two distinct translation mechanism defects

a. Representative immunofluorescence images of control or OSGEP depleted B16F10 treated with ISRIB (15µM) or DMSO. Stress granules (G3BP1) are shown in green and nuclei (DAPI) in blue. **b**. quantification of cells positive to G3BP1 puncta. At least 5 images per conditions were analyzed. **c.** Representative immunofluorescence image of shOSGEP B16F10 showing nuclei (DAPI) in blue, Stress Granule (G3BP1) in green and protein aggregates in red. **d**.

Quantification of protein aggregation (APF) in control or OSGEP depleted B16F10 treated with ISRIB (15µM) or DMSO. Data are reported as mean +/- SD (b,d)). Mann Whitney test (b,d). Exact p-values are indicated. Source data are provided as a Source Data file.

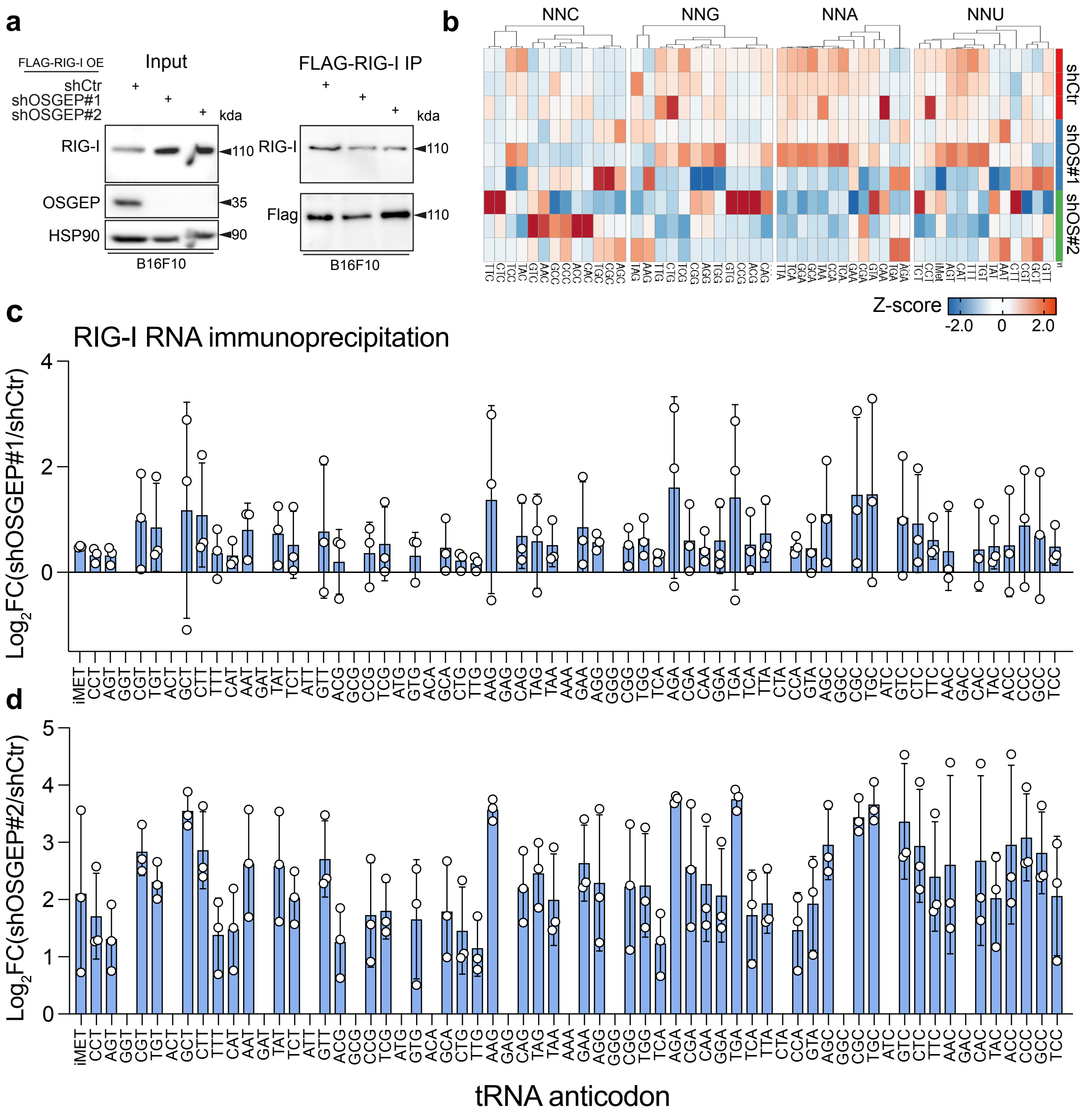

Supplementary figure 13: Analysis of the FLAG-RIG-I RNA immunoprecipitate

a. Western blot from input or FLAG-RIG-I immunoprecipitate for OSGEP, FLAG and RIG-I in B16F10 cells infected with control (Ctr) or OSGEP shRNAs (#1, #2). HSP90 is used loading control. **b-c.** heatmap (a) and Log 2-fold change (b) of tRNAs differentially bound to RIG-I upon depletion of OSGEP using shRNAs (shOS#1, shOS#2), compared to shRNA control (shCtr) (n=3 independent experiments). Source data are provided as a Source Data file.

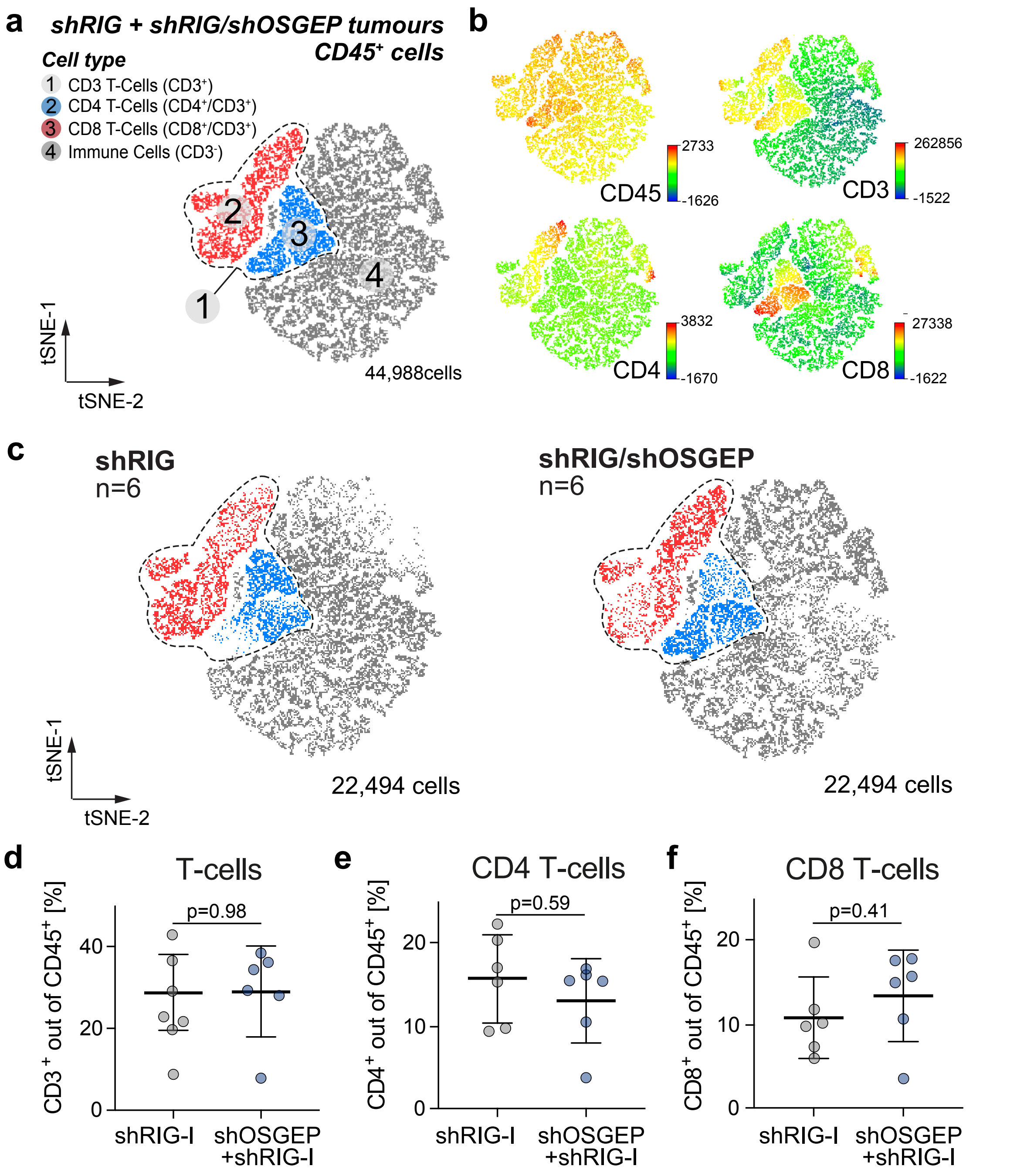

Supplementary figure 14: RIG-I is necessary for T-cell immune infiltration in OSGEP depleted tumours.

a. t-Distributed stochastic neighbor embedding (SNE) representation of T cell population infiltration from 44,988 cells isolated from shRIG and ShRIG/OSGEP tumours (n=3). **b**. t-SNE representation of CD45, CD3, CD4 and CD8 population in B16F10 tumours depleted for shRIG or shRIG/OSGEP. **c.** as in a, but shRIG and shRIG/shOSGEP conditions shown independently (22,494 cells each). **d.** Quantification of FACS analysis of $CD3^+$ T cells in

B16F10 shOSGEP and shRIG/OSGEP tumours. **e**. Quantification of FACS analysis of $CD4^+$ T cells in B16F10 shOSGEP and shRIG/OSGEP tumours. **f**. Quantification of FACS analysis of $CD8^+$ T cell in B16F10 shOSGEP and shRIG/OSGEP tumours. Data are reported as mean +/- SD (d,e,f). Unpaired two-tailed t-test (d,f). Mann Whitney test (e). Exact p-values are indicated. Source data are provided as a Source Data file.

Uncropped Western Blots of Supplementary Figures

Supplementary figure 7d

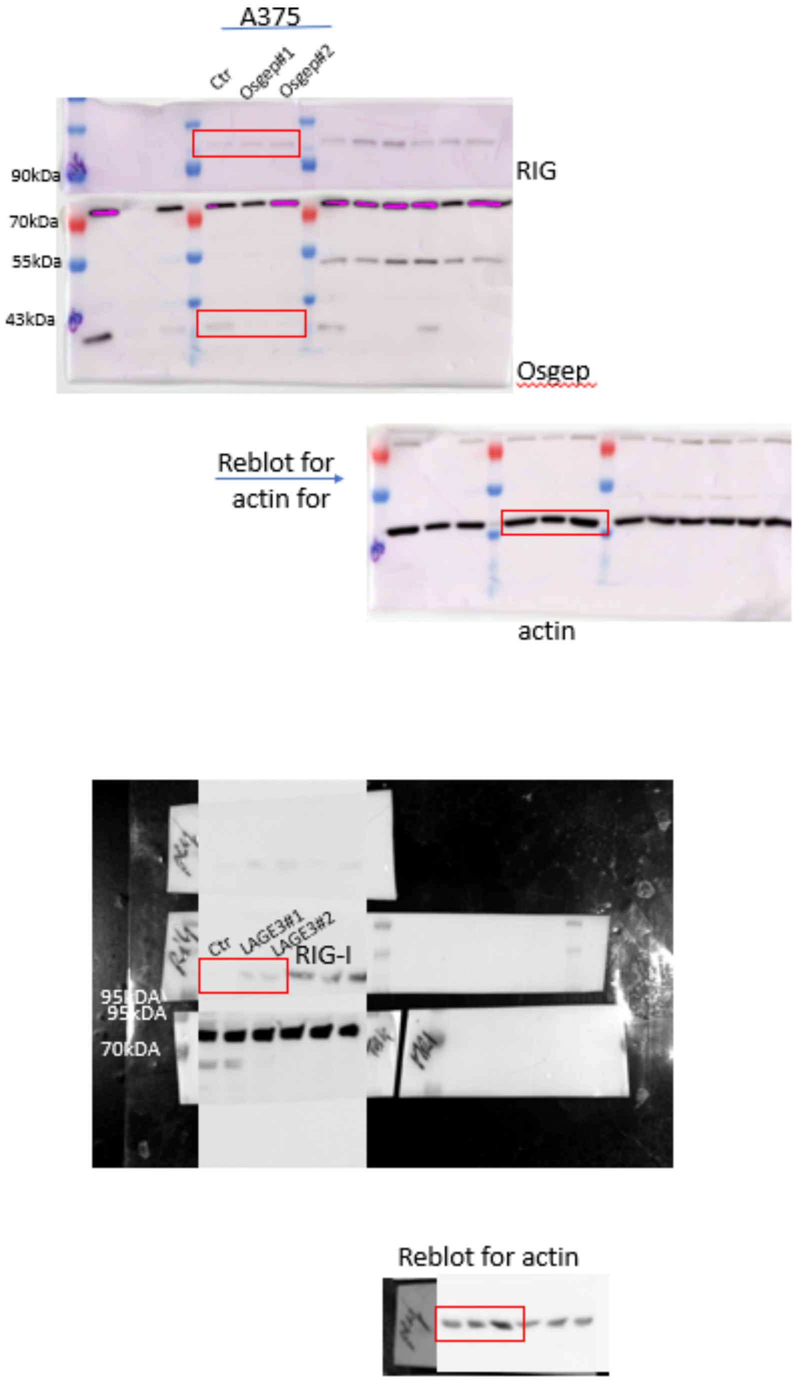

Supplementary figure 7f

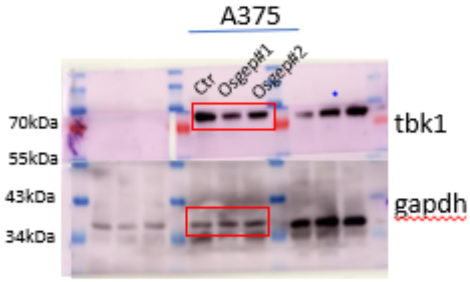

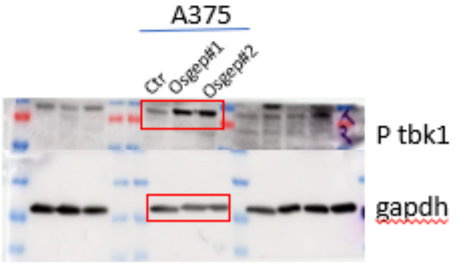

Supplementary figure 7g

7g

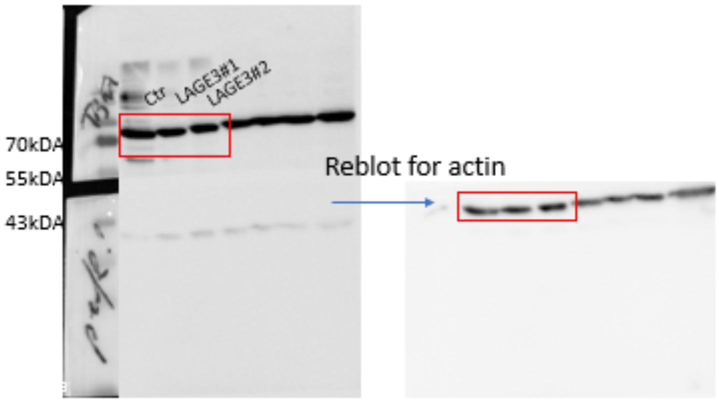

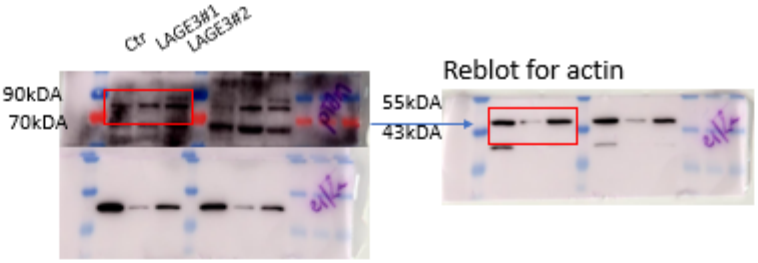

Supplementary figure 11a

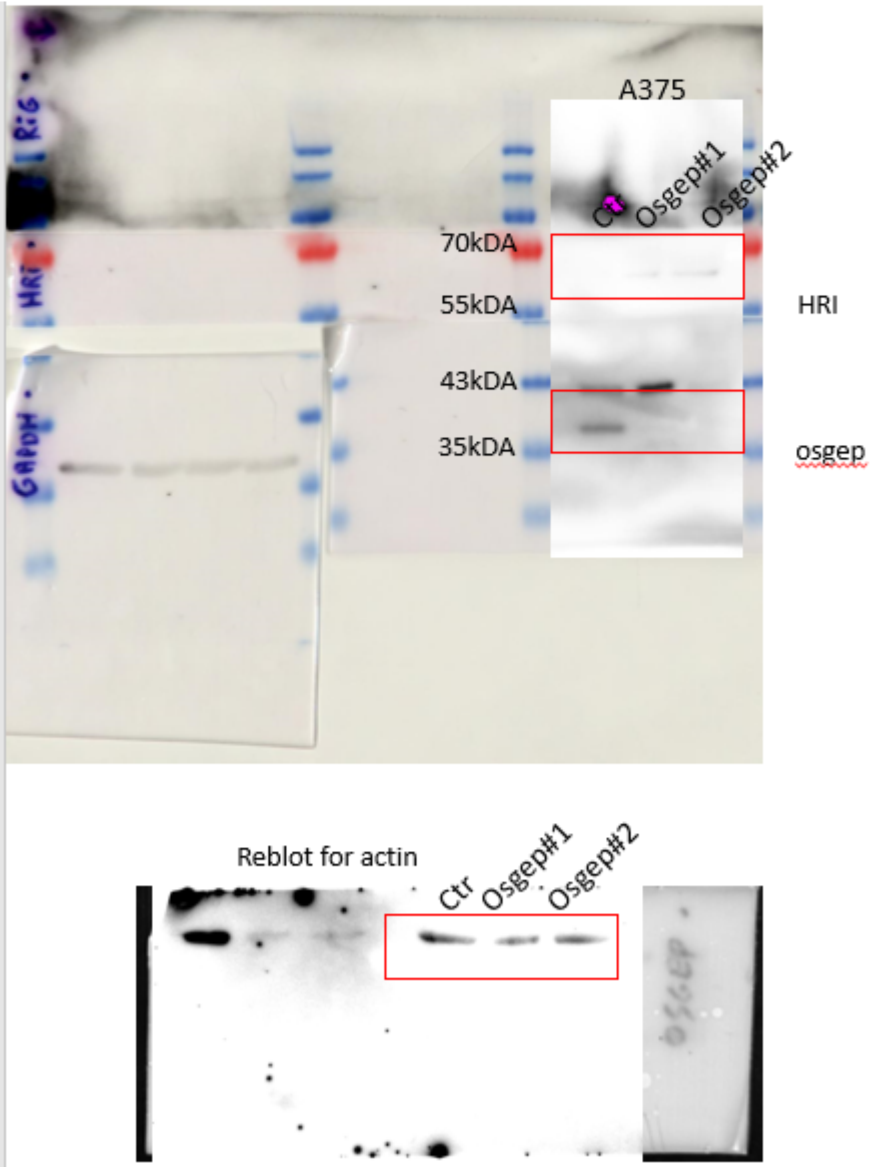

Supplementary figure 11b

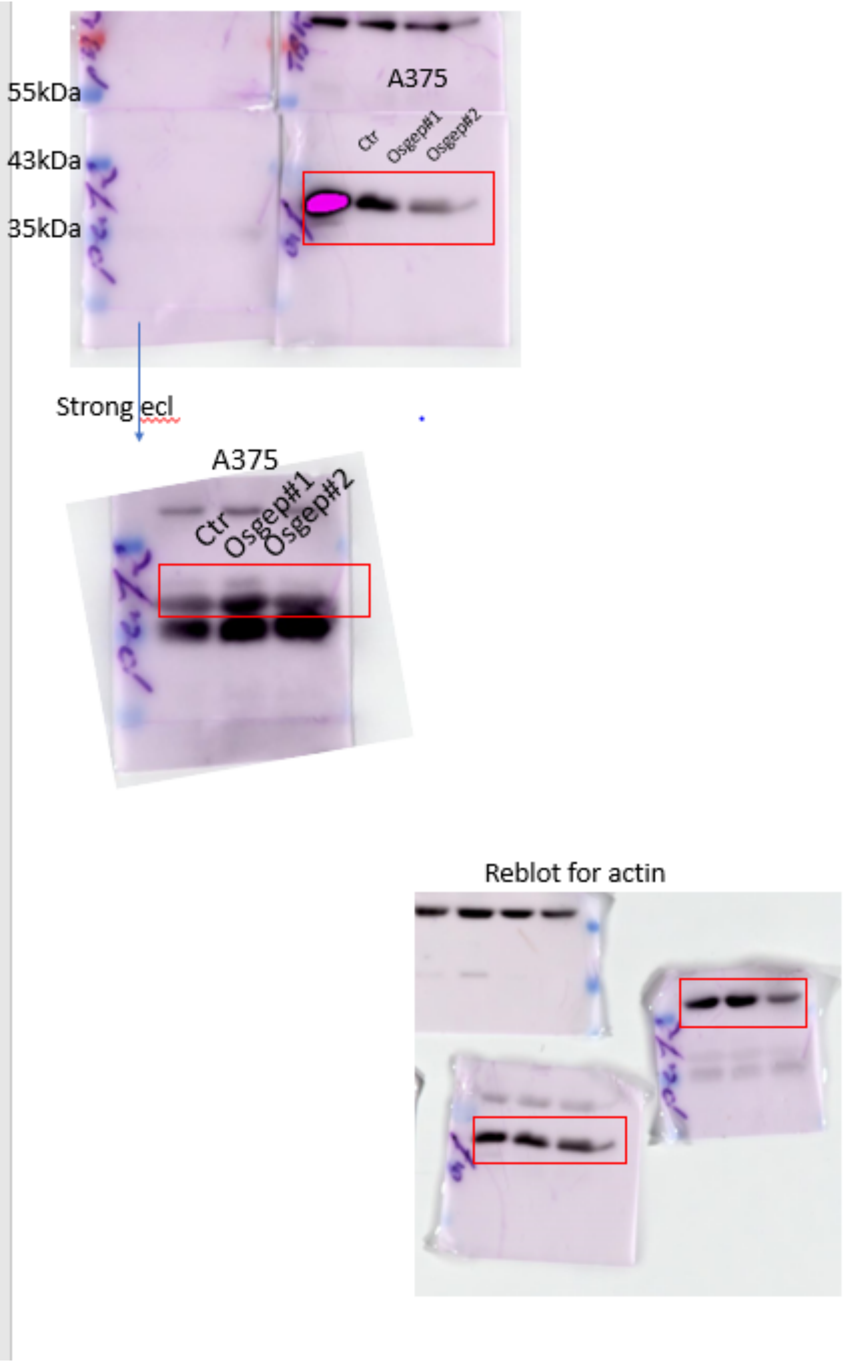

Supplementary figure 11c

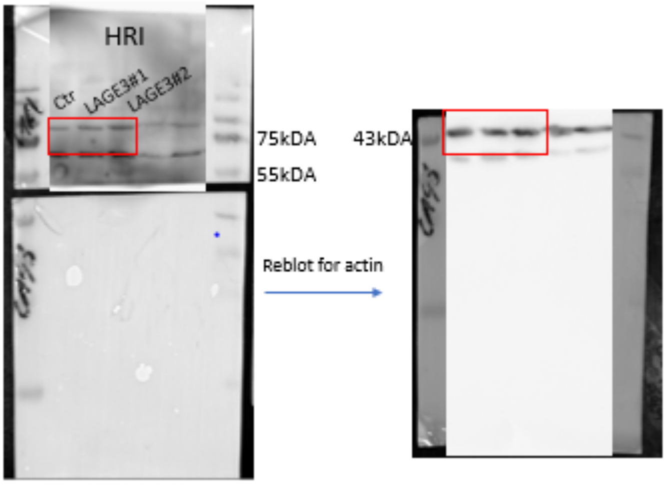

Supplementary figure 11d

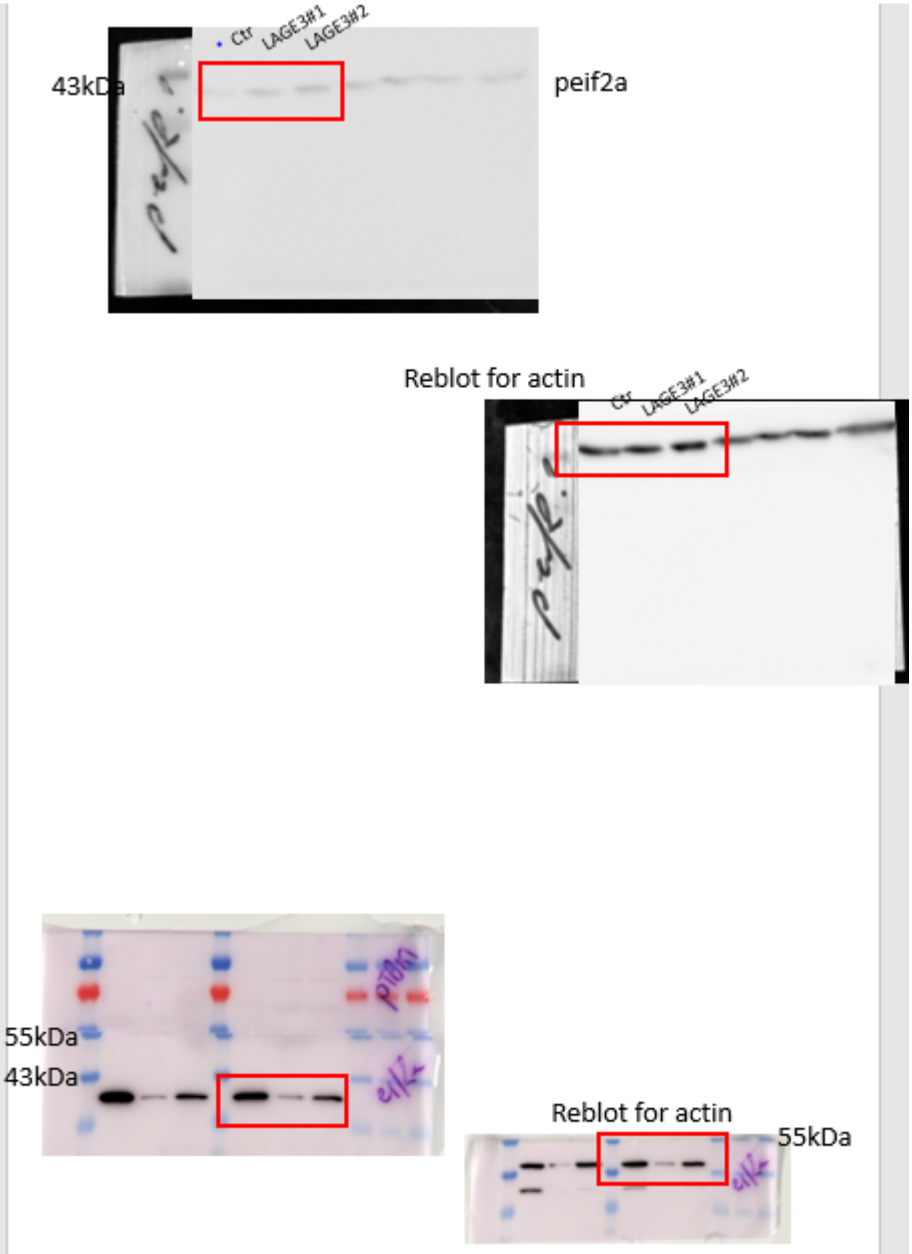

Supplementary figure 11e

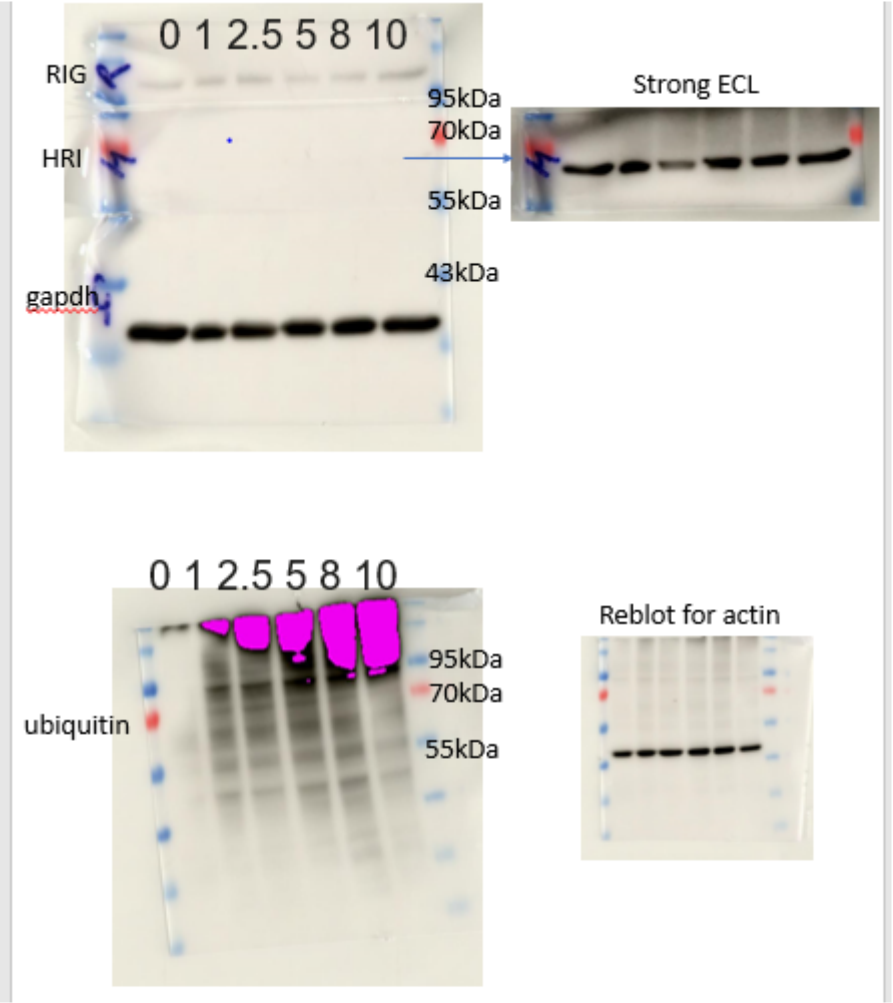

Supplementary figure 11f

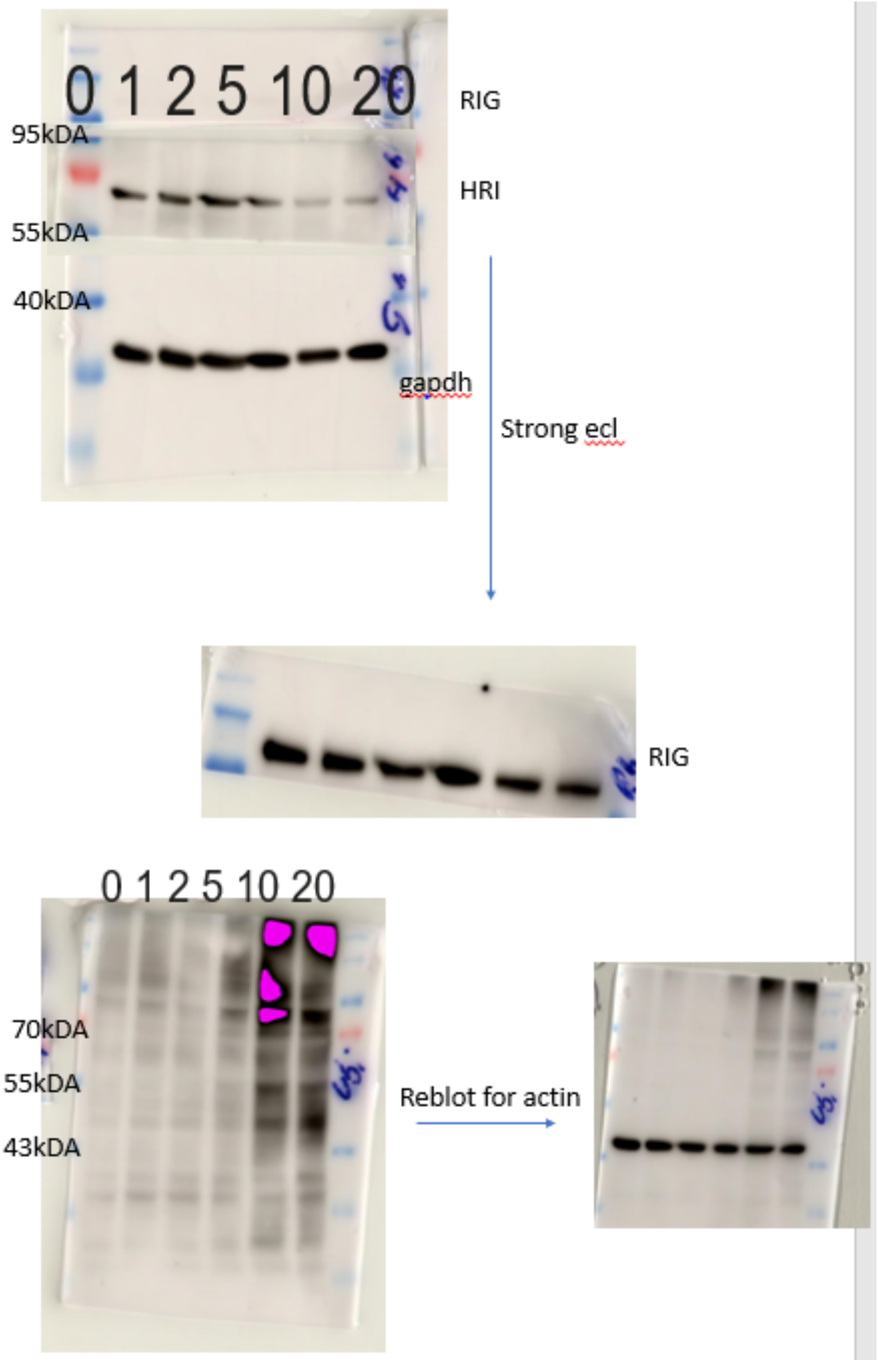

Supplementary figure 11g

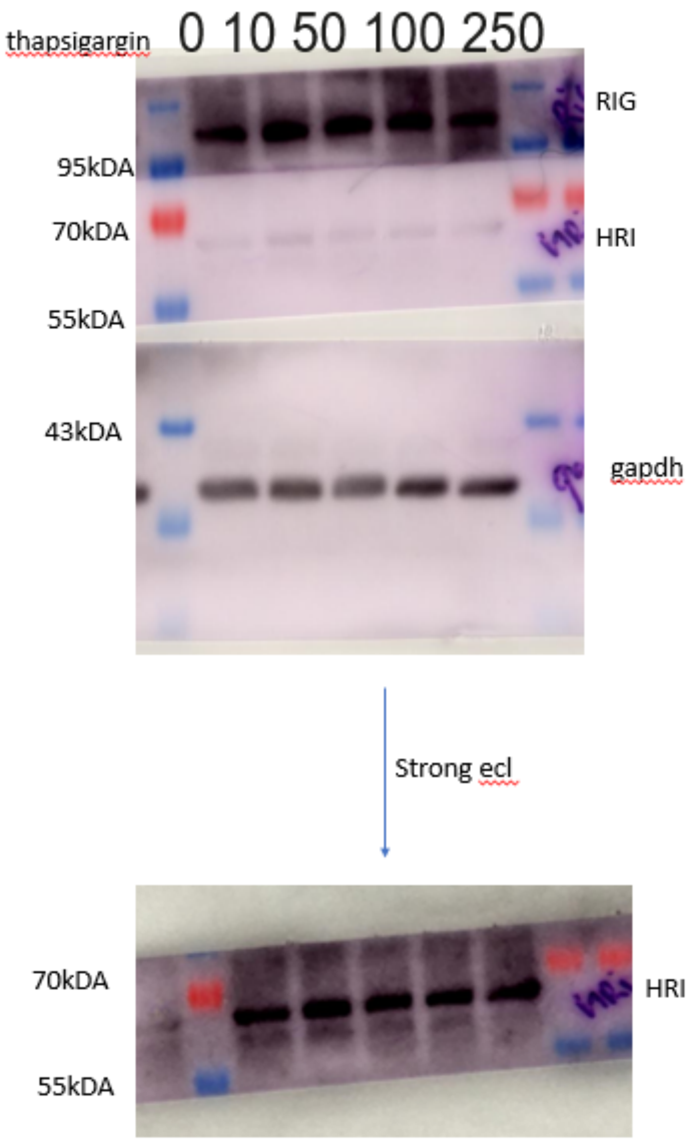

Supplementary figure 11h

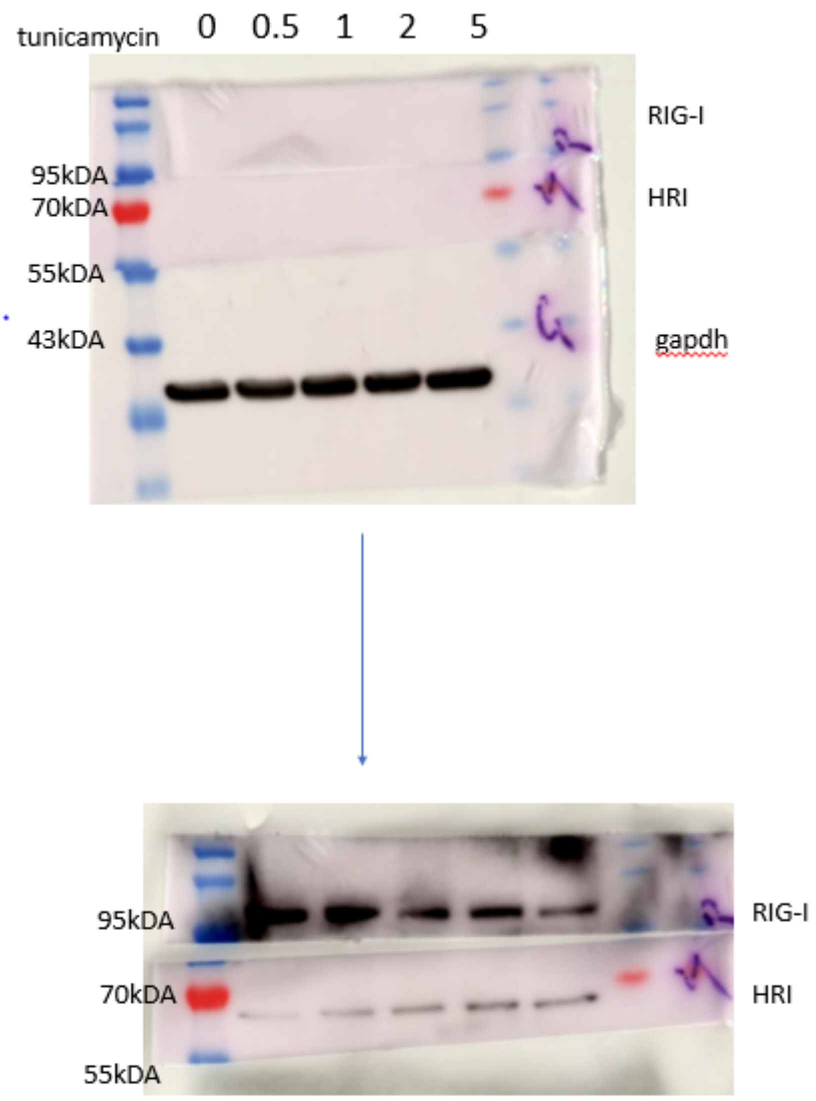

Supplementary figure 12e

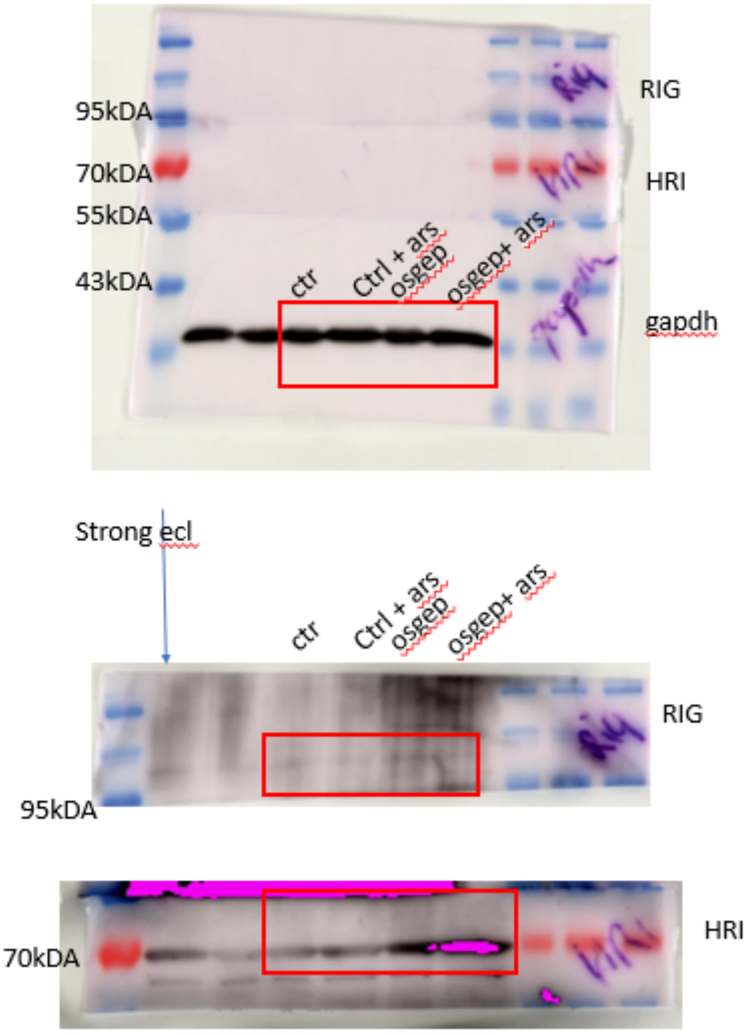

Supplement: Supplementary file 1 — Supplementary information [file 41467_2026_69964_MOESM1_ESM.pdf]
