# Supplementary material for: Disruption of tRNA threonylation triggers RIG-I mediated anti-tumour immune response
Source: Nat Commun. 2026 Feb 25;17:3145. doi: 10.1038/s41467-026-69964-2 (PMC13043769; doi:10.1038/s41467-026-69964-2)
Supplement: Supplementary file 2 — Description of Additional Supplementary Files [file 41467_2026_69964_MOESM2_ESM.pdf]

Description of Additional Supplementary Files

Supplementary Dataset 1: TPA expression in B16F10 melanoma cell line transduced with shRNA targeting OSGEP compared to control shRNA. pValue using the t-test are shown.

Supplementary Dataset 2: Significant MSigDB terms for DEGs among shRNA targeting OSGEP versus shRNA CTR in B16F10 melanoma cell line. p-value: Random permutations (1000x).

Supplementary Dataset 3: levels of tRNA modifications in B16F10 melanoma cells transduced with shRNA#2 targeting OSGEP compared to control shRNA. p-value using the t-test are shown.

Supplementary Dataset 4: Quantitative proteomics of extracted aggregates B16F10 cells infected with shRNA targeting CTR or OSGEP.

Supplementary Dataset 5: Proteins uniquely found in shCTR or shOSGEP B16F10 cells protein aggregates, and list of 78 proteins selected.

Supplementary Dataset 6: Log2FC ribosome codon occupancy of B16F10 cells transduced with shRNA targeting OSGEP or CTR (E, P, A, 1, 2, 3 = translational site of the ribosome).

Supplementary Dataset 7: Reagents used in this study.
