# Supplementary material for: Disruption of tRNA threonylation triggers RIG-I mediated anti-tumour immune response
Source: Nat Commun. 2026 Feb 25;17:3145. doi: 10.1038/s41467-026-69964-2 (PMC13043769; doi:10.1038/s41467-026-69964-2)
Supplement: Supplementary file 10 — Reporting Summary [file 41467_2026_69964_MOESM10_ESM.pdf]

nature portfolio

Corresponding author(s): Pierre CLOSE

Last updated by author(s): 06/02/2026

Reporting Summary

Nature Portfolio wishes to improve the reproducibility of the work that we publish. This form provides structure for consistency and transparency in reporting. For further information on Nature Portfolio policies, see our Editorial Policies and the Editorial Policy Checklist.

Statistics

For all statistical analyses, confirm that the following items are present in the figure legend, table legend, main text, or Methods section.

n/a	Confirmed	
☐	☒	The exact sample size (*n*) for each experimental group/condition, given as a discrete number and unit of measurement
☐	☒	A statement on whether measurements were taken from distinct samples or whether the same sample was measured repeatedly
☐	☒	The statistical test(s) used AND whether they are one- or two-sided<br>*Only common tests should be described solely by name; describe more complex techniques in the Methods section.*
☐	☒	A description of all covariates tested
☐	☒	A description of any assumptions or corrections, such as tests of normality and adjustment for multiple comparisons
☐	☒	A full description of the statistical parameters including central tendency (e.g. means) or other basic estimates (e.g. regression coefficient) AND variation (e.g. standard deviation) or associated estimates of uncertainty (e.g. confidence intervals)
☐	☒	For null hypothesis testing, the test statistic (e.g. F, t, r) with confidence intervals, effect sizes, degrees of freedom and P value noted<br>*Give P values as exact values whenever suitable.*
☒	☐	For Bayesian analysis, information on the choice of priors and Markov chain Monte Carlo settings
☒	☐	For hierarchical and complex designs, identification of the appropriate level for tests and full reporting of outcomes
☒	☐	Estimates of effect sizes (e.g. Cohen's *d*, Pearson's *r*), indicating how they were calculated

Our web collection on statistics for biologists contains articles on many of the points above.

Software and code

Policy information about availability of computer code

Data collection	For ribosome profiling : Mus musculus transcriptome was retrieved (GRCm39) in ENSEMBL- For RNA-seq : Mus musculus genome was retrieved (GRCm38) in ENSEMBL- Proteomics : SwissProt database limited to Mus Musculus taxonomy (17038 sequences, June 2020) - tRNA analysis in RIG-IP : GRCm39, tRNAs reference genome (GRCm39) obtained from the Genomic tRNA database (GtRNAdb 2.0)

Data analysis

Open-source softwares such as STAR, DESeq2 have been used to analyse omics data. See extensive list in the 'Code availability section'". Python (v.3.8.1), bash and R languages have been used to integrate these tools for the dedicated analysis. Statistical analysis have been performed using GraphPad .
Code availability
TrimGalore ! https://www.bioinformatics.babraham.ac.uk/projects/trim_galore/
Bismark (version 0.22.3) https://www.bioinformatics.babraham.ac.uk/projects/bismark/
RSamtools https://bioconductor.org/packages/release/bioc/html/Rsamtools.html
GenomicAlignments https://bioconductor.org/packages/release/bioc/html/GenomicAlignments.html
Pysam https://github.com/pysam-developers/pysam

ToppGene https://toppgene.cchmc.org/
FASTX-Toolkit http://hannonlab.cshl.edu/fastx_toolkit/
STAR https://github.com/alexdobin/STAR
DESeq2 https://bioconductor.org/packages/release/bioc/html/DESeq2.html
EdgeR https://bioconductor.org/packages/release/bioc/html/edgeR.html
GSVA https://www.bioconductor.org/packages/release/bioc/html/GSVA.html

For manuscripts utilizing custom algorithms or software that are central to the research but not yet described in published literature, software must be made available to editors and reviewers. We strongly encourage code deposition in a community repository (e.g. GitHub). See the Nature Portfolio guidelines for submitting code & software for further information.

Data

Policy information about availability of data

All manuscripts must include a data availability statement. This statement should provide the following information, where applicable:

- Accession codes, unique identifiers, or web links for publicly available datasets
- A description of any restrictions on data availability
- For clinical datasets or third party data, please ensure that the statement adheres to our policy

Proteomics data: The mass spectrometry data have been deposited in ProteomeXchange with the primary accession code : PXD059835
Ribosome sequencing: Gene Expression Omnibus; accession number : GSE286704
RNA sequencing: Gene Expression Omnibus; GSE286709
RNA sequencing after IP-RIG : Gene Expression Omnibus; GSE286715
Spatial transcriptomic dataset: GSE316760

Research involving human participants, their data, or biological material

Policy information about studies with human participants or human data. See also policy information about sex, gender (identity/presentation), and sexual orientation and race, ethnicity and racism.

Reporting on sex and gender	sex and gender was not considered in our study
Reporting on race, ethnicity, or other socially relevant groupings	race, ethnicity and other socially relevant groupings were not considered
Population characteristics	human samples were patient presenting cutaneous melanoma
Recruitment	human samples were patient presenting cutaneous melanoma. No specific other selection criteria was applied.
Ethics oversight	The human study sample project was reviewed and approved by Research Ethics Committee and NHS Health Research Authority (IRAS project ID: 216310 REC reference: 16/LO/2098), sponsored by the University of Manchester.

Note that full information on the approval of the study protocol must also be provided in the manuscript.

Field-specific reporting

Please select the one below that is the best fit for your research. If you are not sure, read the appropriate sections before making your selection.

☒ Life sciences ☐ Behavioural & social sciences ☐ Ecological, evolutionary & environmental sciences

For a reference copy of the document with all sections, see nature.com/documents/nr-reporting-summary-flat.pdf

Life sciences study design

All studies must disclose on these points even when the disclosure is negative.

Sample size

Animal sample size experiment was assessed by Web Power. For the other experiments, no sample size calculation was performed.

Data exclusions	Concerning mice experiment, we performed ROUT test on GraphPad Prism to identify outliers
Replication	All experiments were performed with at least 2 biological replicates. The exact number of replicates is stated in figure legends
Randomization	Randomization was not performed, experiments were done in cell lines or xenografts were randomization is not applicable.
Blinding	Xenograft experiments were not performed in blind as these experiments are conducted for explorative purposes.

Behavioural & social sciences study design

All studies must disclose on these points even when the disclosure is negative.

Study description	*Briefly describe the study type including whether data are quantitative, qualitative, or mixed-methods (e.g. qualitative cross-sectional, quantitative experimental, mixed-methods case study).*
Research sample	*State the research sample (e.g. Harvard university undergraduates, villagers in rural India) and provide relevant demographic information (e.g. age, sex) and indicate whether the sample is representative. Provide a rationale for the study sample chosen. For studies involving existing datasets, please describe the dataset and source.*
Sampling strategy	*Describe the sampling procedure (e.g. random, snowball, stratified, convenience). Describe the statistical methods that were used to predetermine sample size OR if no sample-size calculation was performed, describe how sample sizes were chosen and provide a rationale for why these sample sizes are sufficient. For qualitative data, please indicate whether data saturation was considered, and what criteria were used to decide that no further sampling was needed.*
Data collection	*Provide details about the data collection procedure, including the instruments or devices used to record the data (e.g. pen and paper, computer, eye tracker, video or audio equipment) whether anyone was present besides the participant(s) and the researcher, and whether the researcher was blind to experimental condition and/or the study hypothesis during data collection.*
Timing	*Indicate the start and stop dates of data collection. If there is a gap between collection periods, state the dates for each sample cohort.*
Data exclusions	*If no data were excluded from the analyses, state so OR if data were excluded, provide the exact number of exclusions and the rationale behind them, indicating whether exclusion criteria were pre-established.*
Non-participation	*State how many participants dropped out/declined participation and the reason(s) given OR provide response rate OR state that no participants dropped out/declined participation.*
Randomization	*If participants were not allocated into experimental groups, state so OR describe how participants were allocated to groups, and if allocation was not random, describe how covariates were controlled.*

Ecological, evolutionary & environmental sciences study design

All studies must disclose on these points even when the disclosure is negative.

Study description	*Briefly describe the study. For quantitative data include treatment factors and interactions, design structure (e.g. factorial, nested, hierarchical), nature and number of experimental units and replicates.*
Research sample	*Describe the research sample (e.g. a group of tagged Passer domesticus, all Stenocereus thurberi within Organ Pipe Cactus National Monument), and provide a rationale for the sample choice. When relevant, describe the organism taxa, source, sex, age range and any manipulations. State what population the sample is meant to represent when applicable. For studies involving existing datasets, describe the data and its source.*
Sampling strategy	*Note the sampling procedure. Describe the statistical methods that were used to predetermine sample size OR if no sample-size calculation was performed, describe how sample sizes were chosen and provide a rationale for why these sample sizes are sufficient.*
Data collection	*Describe the data collection procedure, including who recorded the data and how.*
Timing and spatial scale	*Indicate the start and stop dates of data collection, noting the frequency and periodicity of sampling and providing a rationale for these choices. If there is a gap between collection periods, state the dates for each sample cohort. Specify the spatial scale from which the data are taken*
Data exclusions	*If no data were excluded from the analyses, state so OR if data were excluded, describe the exclusions and the rationale behind them, indicating whether exclusion criteria were pre-established.*
Reproducibility	*Describe the measures taken to verify the reproducibility of experimental findings. For each experiment, note whether any attempts to repeat the experiment failed OR state that all attempts to repeat the experiment were successful.*
Randomization	*Describe how samples/organisms/participants were allocated into groups. If allocation was not random, describe how covariates were controlled. If this is not relevant to your study, explain why.*

Blinding | *Describe the extent of blinding used during data acquisition and analysis. If blinding was not possible, describe why OR explain why blinding was not relevant to your study.*

Did the study involve field work? ☐ Yes ☐ No

Field work, collection and transport

Field conditions	*Describe the study conditions for field work, providing relevant parameters (e.g. temperature, rainfall).*
Location	*State the location of the sampling or experiment, providing relevant parameters (e.g. latitude and longitude, elevation, water depth).*
Access & import/export	*Describe the efforts you have made to access habitats and to collect and import/export your samples in a responsible manner and in compliance with local, national and international laws, noting any permits that were obtained (give the name of the issuing authority, the date of issue, and any identifying information).*
Disturbance	*Describe any disturbance caused by the study and how it was minimized.*

Reporting for specific materials, systems and methods

We require information from authors about some types of materials, experimental systems and methods used in many studies. Here, indicate whether each material, system or method listed is relevant to your study. If you are not sure if a list item applies to your research, read the appropriate section before selecting a response.

Materials & experimental systems

n/a	Involved in the study	
☐	☒	Antibodies
☐	☒	Eukaryotic cell lines
☒	☐	Palaeontology and archaeology
☐	☒	Animals and other organisms
☒	☐	Clinical data
☒	☐	Dual use research of concern
☒	☐	Plants

Methods

n/a	Involved in the study	
☒	☐	ChIP-seq
☐	☒	Flow cytometry
☒	☐	MRI-based neuroimaging

Antibodies

Antibodies used

All antibodies and their dilutions used in this study are listed in the supplementary table 6 : Anti-β-Actin–Peroxidase, Alpha-Tubulin mono clone B-5-1-2, cGAS (D1D3G) ,eIF2 alpha Phospho-eIF2 alpha (Ser 51), GAPDH Antibody (FL-335), HRI (D-12), Hsp90 alpha/beta (H-114), IRF-3 (FL-425) ,MDA-5 (D74E4), OSGEP ,Phospho-STING (Ser365) (D8F4W), P-TBK1/NAK (S172) (D52C2) XP (R) ,Rig-I (D14G6) ,STING (D2P2F) ,TBK1/NAK ,ECL™ anti-mouse IgG, HRP-linked whole Ab ECL™, anti-rabbit IgG, HRP-linked whole Ab ,IRF-3 (FL-425), DAPI CD3-epsilon sp7, Rig-I (D14G6) ,G3BP1, Monoclonal Proteostat® Aggresome, KI-67 ,Cleaved Caspase-3 (Asp147, 5A1E), RIG-1 ,CD45 CD8, CD3, CD4, CD19 ,F4/80, Ly-6G, NK1.1, TNFa, INFg ,7AAD

Here the table
actin Anti-β-Actin–Peroxidase rabbit sigma A3854 WB 1/10000
a-TUBULIN Alpha-Tubulin mono clone B-5-1-2 Mouse Sigma T6074 WB 1/1000
cGAS cGAS (D1D3G) rabbit cell signaling 15102S WB 1/1000
eIF2 alpha eIF2 alpha rabbit cell signaling 9722 WB 1/1000
eIF2 alpha (P) Phospho-eIF2 alpha (Ser 51) rabbit cell signaling 99721s WB 1/800
GAPDH GAPDH Antibody (FL-335) Rabbit Santa Cruz sc-25778 WB 1/1000
HRI HRI (D-12) mouse santa cruz sc-365239 WB 1/1000
HSP90 Hsp90 alpha/beta (H-114) Rabbit Santa-Cruz sc-7947 WB 1/1000
IRF-3 IRF-3 (FL-425) rabbit santa cruz sc-9082 WB 1/800
MDA-5 MDA-5 (D74E4) rabbit cell signaling 5321S WB 1/1000
OSGEP OSGEP mouse novusbio NBP2-00823 WB 1/800
p-STING Phospho-STING (Ser365) (D8F4W) rabbit cell signaling 72971S WB 1/1000
p-TBK1 P-TBK1/NAK (S172) (D52C2) XP (R) Rabbit cell signaling 5483S WB 1/800
RIG-1 Rig-I (D14G6) rabbit cell signaling 3743T WB 1/1000
STING STING (D2P2F) rabbit cell signaling 13647S WB 1/1000
TBK1 TBK1/NAK rabbit cell signaling 3013S WB 1/800
mouse secondary ECL™ anti-mouse IgG, HRP-linked whole Ab Sheep CiteAb NA931V WB 1/3000
rabbit secondary ECL™ anti-rabbit IgG, HRP-linked whole Ab Sheep CiteAb NA934V WB 1/3000
IRF3 IRF-3 (FL-425) rabbit santa cruz sc-9082 IF 1/200
DAPI DAPI N/A sigma D9542 IF 1/10000
CD3 CD3-epsilon sp7 rabbit abcam ab16669 IF 1/200

RIG Rig-I (D14G6) rabbit Invitrogen 700366 IF 1/100
G3BP1 G3BP1 Monoclonal mouse Proteintech 66486-1-Ig IF 1/250
Aggregats Proteostat® Aggresome N/A ENZO PADR-A10C IF 1/1000
KI-67 KI-67 mouse BD Biosciences 550609 IHC 1/200
Cleaved Caspase-3 Cleaved Caspase-3 (Asp147, 5A1E) rabbit Cell Signaling 9661 IHC 1/400
RIG-1 RIG-1 rabbit Invitrogen 700366 IHC 1/100
CD45 CD45 mouse Milteny 130-110-665 Flow 1/50
CD8 CD8 mouse Milteny 130-119-123 Flow 1/50
CD3 CD3 mouse Milteny 130-119-793 Flow 1/50
CD4 CD4 mouse Milteny 130-110-802 Flow 1/50
B cells CD19 mouse Milteny 130-111-888 Flow 1/50
Macrophages F4/80 mouse Milteny 130-116-499 Flow 1/50
Neutrophiles Ly-6G mouse Milteny 130-121-438 Flow 1/50
NK NK1.1 mouse Milteny 130-120-507 Flow 1/50
TNFa TNFa mouse Milteny 130-119-561 Flow 1/50
INFg INFg mouse Milteny 130-123-283 Flow 1/50
7AAD 7AAD mouse Milteny 130-111-568 Flow 1/50

Validation

Validation of the listed antibodies was performed by the manufacturer.

Eukaryotic cell lines

Policy information about cell lines and Sex and Gender in Research

Cell line source(s)

Cell lines source is stated in the material and methods section of the manuscript (Cell culture). B16 were grown in Dulbecco's modified Eagle's medium (DMEM, LONZA) supplemented with 1% glutamine and 1% penicillin-streptomycin and 10% FBS (GIBCO). M1014 were obtained from Lionel Larue (Curie Institute), MM117 and MM011 were provided by were from Pr. G. Ghanem (Institut J. Bordet, Université Libre de Bruxelles)and were grown in HAM's medium supplemented with 10% FBS and 1% penicillin-streptomycin

Authentication

None of the cell lines used were authenticated.

Mycoplasma contamination

Mycoplasma test was performed routinely. Only negative lines are used in the study.

Commonly misidentified lines
(See ICLAC register)

None of the used cells are in the misidentified lines.

Palaeontology and Archaeology

Specimen provenance

Provide provenance information for specimens and describe permits that were obtained for the work (including the name of the issuing authority, the date of issue, and any identifying information). Permits should encompass collection and, where applicable, export.

Specimen deposition

Indicate where the specimens have been deposited to permit free access by other researchers.

Dating methods

If new dates are provided, describe how they were obtained (e.g. collection, storage, sample pretreatment and measurement), where they were obtained (i.e. lab name), the calibration program and the protocol for quality assurance OR state that no new dates are provided.

☐ Tick this box to confirm that the raw and calibrated dates are available in the paper or in Supplementary Information.

Ethics oversight

Identify the organization(s) that approved or provided guidance on the study protocol, OR state that no ethical approval or guidance was required and explain why not.

Note that full information on the approval of the study protocol must also be provided in the manuscript.

Animals and other research organisms

Policy information about studies involving animals; ARRIVE guidelines recommended for reporting animal research, and Sex and Gender in Research

Laboratory animals

Female C57BL/6 and NOD-SCID N (impaired T and B cells, defective NK) were obtained from Charles River. Mice were 6-8 weeks old at the time of the tumour inoculation

Wild animals

no wild animal was used in thids study

Reporting on sex

Sex was not considered in the study design.

Field-collected samples

no field collected samples were used in the study.

Ethics oversight

All mouse husbandry and experiments were carried out according to the local ethics committee ULiège under the terms and

Ethics oversight	conditions of the animal licence #2268.

Note that full information on the approval of the study protocol must also be provided in the manuscript.

Clinical data

Policy information about clinical studies

All manuscripts should comply with the ICMJE guidelines for publication of clinical research and a completed CONSORT checklist must be included with all submissions.

Clinical trial registration	*Provide the trial registration number from ClinicalTrials.gov or an equivalent agency.*
Study protocol	*Note where the full trial protocol can be accessed OR if not available, explain why.*
Data collection	*Describe the settings and locales of data collection, noting the time periods of recruitment and data collection.*
Outcomes	*Describe how you pre-defined primary and secondary outcome measures and how you assessed these measures.*

Dual use research of concern

Policy information about dual use research of concern

Hazards

Could the accidental, deliberate or reckless misuse of agents or technologies generated in the work, or the application of information presented in the manuscript, pose a threat to:

No	Yes	
☒	☐	Public health
☒	☐	National security
☒	☐	Crops and/or livestock
☒	☐	Ecosystems
☒	☐	Any other significant area

Experiments of concern

Does the work involve any of these experiments of concern:

No	Yes	
☒	☐	Demonstrate how to render a vaccine ineffective
☒	☐	Confer resistance to therapeutically useful antibiotics or antiviral agents
☒	☐	Enhance the virulence of a pathogen or render a nonpathogen virulent
☒	☐	Increase transmissibility of a pathogen
☒	☐	Alter the host range of a pathogen
☒	☐	Enable evasion of diagnostic/detection modalities
☒	☐	Enable the weaponization of a biological agent or toxin
☒	☐	Any other potentially harmful combination of experiments and agents

Plants

Seed stocks	*Report on the source of all seed stocks or other plant material used. If applicable, state the seed stock centre and catalogue number. If plant specimens were collected from the field, describe the collection location, date and sampling procedures.*
Novel plant genotypes	*Describe the methods by which all novel plant genotypes were produced. This includes those generated by transgenic approaches, gene editing, chemical/radiation-based mutagenesis and hybridization. For transgenic lines, describe the transformation method, the number of independent lines analyzed and the generation upon which experiments were performed. For gene-edited lines, describe the editor used, the endogenous sequence targeted for editing, the targeting guide RNA sequence (if applicable) and how the editor was applied.*
Authentication	*Describe any authentication procedures for each seed stock used or novel genotype generated. Describe any experiments used to assess the effect of a mutation and, where applicable, how potential secondary effects (e.g. second site T-DNA insertions, mosiacism, off-target gene editing) were examined.*

ChIP-seq

Data deposition

☐ Confirm that both raw and final processed data have been deposited in a public database such as GEO.

☐ Confirm that you have deposited or provided access to graph files (e.g. BED files) for the called peaks.

Data access links<br>*May remain private before publication.*	*For "Initial submission" or "Revised version" documents, provide reviewer access links. For your "Final submission" document, provide a link to the deposited data.*
Files in database submission	*Provide a list of all files available in the database submission.*
Genome browser session<br>(e.g. UCSC)	*Provide a link to an anonymized genome browser session for "Initial submission" and "Revised version" documents only, to enable peer review. Write "no longer applicable" for "Final submission" documents.*

Methodology

Replicates	*Describe the experimental replicates, specifying number, type and replicate agreement.*
Sequencing depth	*Describe the sequencing depth for each experiment, providing the total number of reads, uniquely mapped reads, length of reads and whether they were paired- or single-end.*
Antibodies	*Describe the antibodies used for the ChIP-seq experiments; as applicable, provide supplier name, catalog number, clone name, and lot number.*
Peak calling parameters	*Specify the command line program and parameters used for read mapping and peak calling, including the ChIP, control and index files used.*
Data quality	*Describe the methods used to ensure data quality in full detail, including how many peaks are at FDR 5% and above 5-fold enrichment.*
Software	*Describe the software used to collect and analyze the ChIP-seq data. For custom code that has been deposited into a community repository, provide accession details.*

Flow Cytometry

Plots

Confirm that:

☒ The axis labels state the marker and fluorochrome used (e.g. CD4-FITC).

☒ The axis scales are clearly visible. Include numbers along axes only for bottom left plot of group (a 'group' is an analysis of identical markers).

☒ All plots are contour plots with outliers or pseudocolor plots.

☒ A numerical value for number of cells or percentage (with statistics) is provided.

Methodology

Sample preparation	Tumors were extracted on day 14 post-injection and dissociated in digestion media (collagenase, FBS, DNAse) with GentleMACS using digestion tumor program mTDK2, 37°C for 45min. Staining was performed according to protocols of Transcription factor, intracellular and cell surface staining from Milteny's manufacturer. Briefly tumours were filtered through 70µM cell strainer and cells were counted. 1,000,000 tumour cells were stained with 7-AAD viability to exclude dead cells, washed and stained with cell surface antibodies. INFα and TNFα staining was performed using Inside Stain kit (Miltenyi #130-090-47), according to manufacturer's instructions. Briefly, cells were fixed and permeabilized with Fixation/ Permeabilization solution during 30 minutes, and stained with the mix of antibodies for 30 minutes in the dark at 4°C.
Instrument	BD FACSCanto™ II.
Software	using FlowJo Software (Version 10).
Cell population abundance	No sorting was performed
Gating strategy	gating strategy are provided in supplementary information. t cells were gated on FSC-SCC, doublet were remove (FSC_A/FSC-H). cell negative for cell death were then gated for CD45. Positive cells to CD45, were gated for CD3. CD3 positive cells were gated for CD4 or CD8.

☒ Tick this box to confirm that a figure exemplifying the gating strategy is provided in the Supplementary Information.

Magnetic resonance imaging

Experimental design

Design type	*Indicate task or resting state; event-related or block design.*
Design specifications	*Specify the number of blocks, trials or experimental units per session and/or subject, and specify the length of each trial or block (if trials are blocked) and interval between trials.*
Behavioral performance measures	*State number and/or type of variables recorded (e.g. correct button press, response time) and what statistics were used to establish that the subjects were performing the task as expected (e.g. mean, range, and/or standard deviation across subjects).*

Acquisition

Imaging type(s)	*Specify: functional, structural, diffusion, perfusion.*
Field strength	*Specify in Tesla*
Sequence & imaging parameters	*Specify the pulse sequence type (gradient echo, spin echo, etc.), imaging type (EPI, spiral, etc.), field of view, matrix size, slice thickness, orientation and TE/TR/flip angle.*
Area of acquisition	*State whether a whole brain scan was used OR define the area of acquisition, describing how the region was determined.*

Diffusion MRI ☐ Used ☐ Not used

Preprocessing

Preprocessing software	*Provide detail on software version and revision number and on specific parameters (model/functions, brain extraction, segmentation, smoothing kernel size, etc.).*
Normalization	*If data were normalized/standardized, describe the approach(es): specify linear or non-linear and define image types used for transformation OR indicate that data were not normalized and explain rationale for lack of normalization.*
Normalization template	*Describe the template used for normalization/transformation, specifying subject space or group standardized space (e.g. original Talairach, MNI305, ICBM152) OR indicate that the data were not normalized.*
Noise and artifact removal	*Describe your procedure(s) for artifact and structured noise removal, specifying motion parameters, tissue signals and physiological signals (heart rate, respiration).*
Volume censoring	*Define your software and/or method and criteria for volume censoring, and state the extent of such censoring.*

Statistical modeling & inference

Model type and settings	*Specify type (mass univariate, multivariate, RSA, predictive, etc.) and describe essential details of the model at the first and second levels (e.g. fixed, random or mixed effects; drift or auto-correlation).*
Effect(s) tested	*Define precise effect in terms of the task or stimulus conditions instead of psychological concepts and indicate whether ANOVA or factorial designs were used.*

Specify type of analysis: ☐ Whole brain ☐ ROI-based ☐ Both

Statistic type for inference<br>(See Eklund et al. 2016)	*Specify voxel-wise or cluster-wise and report all relevant parameters for cluster-wise methods.*
Correction	*Describe the type of correction and how it is obtained for multiple comparisons (e.g. FWE, FDR, permutation or Monte Carlo).*

Models & analysis

n/a	Involved in the study
☐	☐ Functional and/or effective connectivity
☐	☐ Graph analysis
☐	☐ Multivariate modeling or predictive analysis

Functional and/or effective connectivity	*Report the measures of dependence used and the model details (e.g. Pearson correlation, partial correlation, mutual information).*
Graph analysis	*Report the dependent variable and connectivity measure, specifying weighted graph or binarized graph,*

Graph analysis

subject- or group-level, and the global and/or node summaries used (e.g. clustering coefficient, efficiency, etc.).

Multivariate modeling and predictive analysis

Specify independent variables, features extraction and dimension reduction, model, training and evaluation metrics.
