# Supplementary material for: Disruption of tRNA threonylation triggers RIG-I mediated anti-tumour immune response
Source: Nat Commun. 2026 Feb 25;17:3145. doi: 10.1038/s41467-026-69964-2 (PMC13043769; doi:10.1038/s41467-026-69964-2)

Gel from Western Blot FIGURE 3E

B16 PANEL

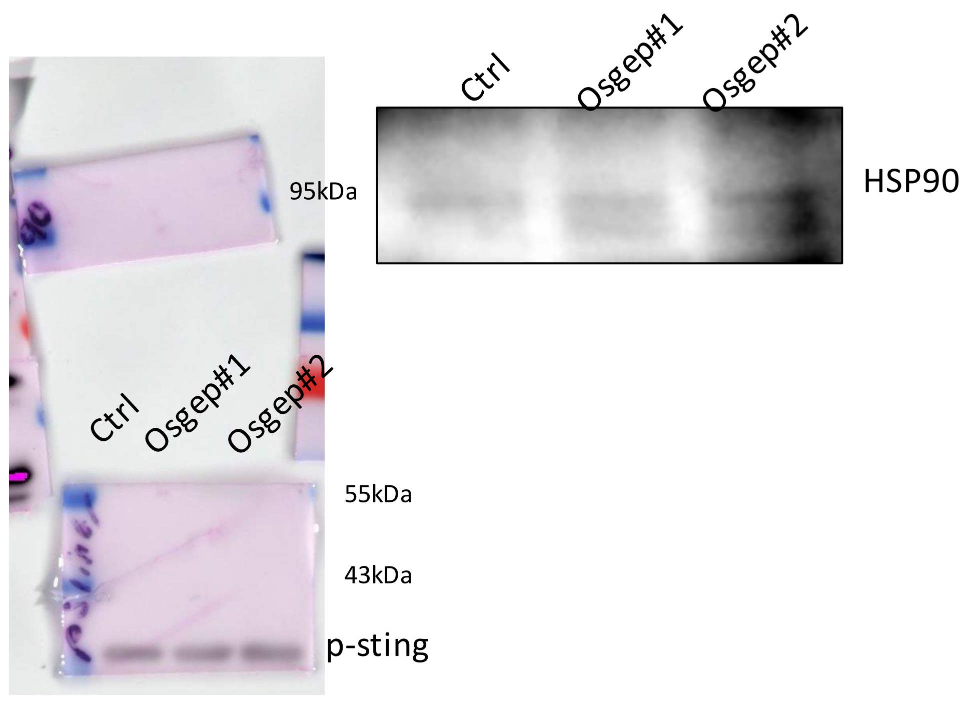

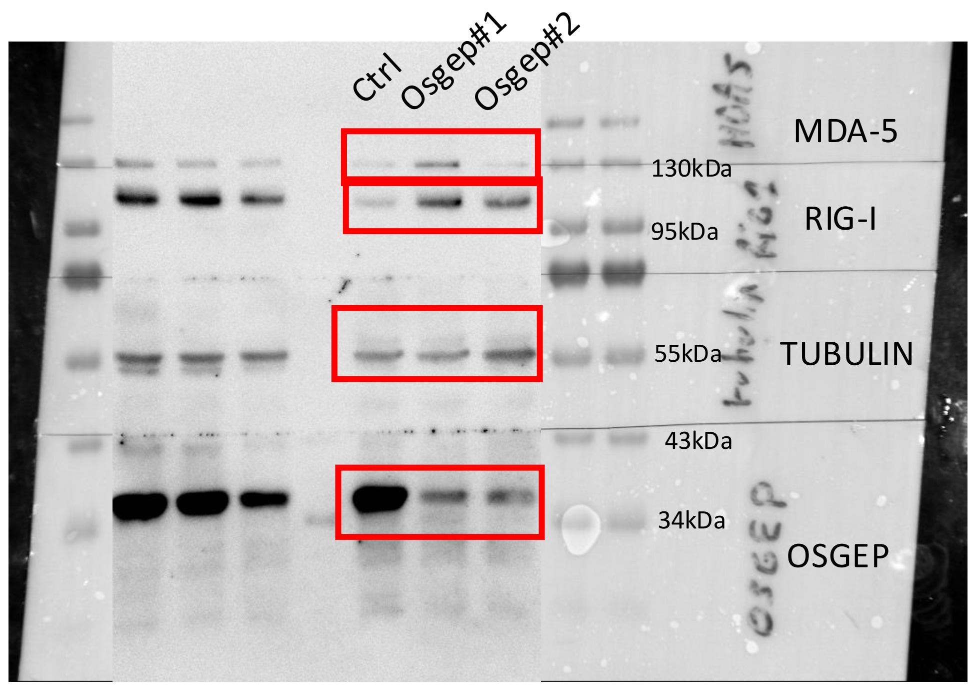

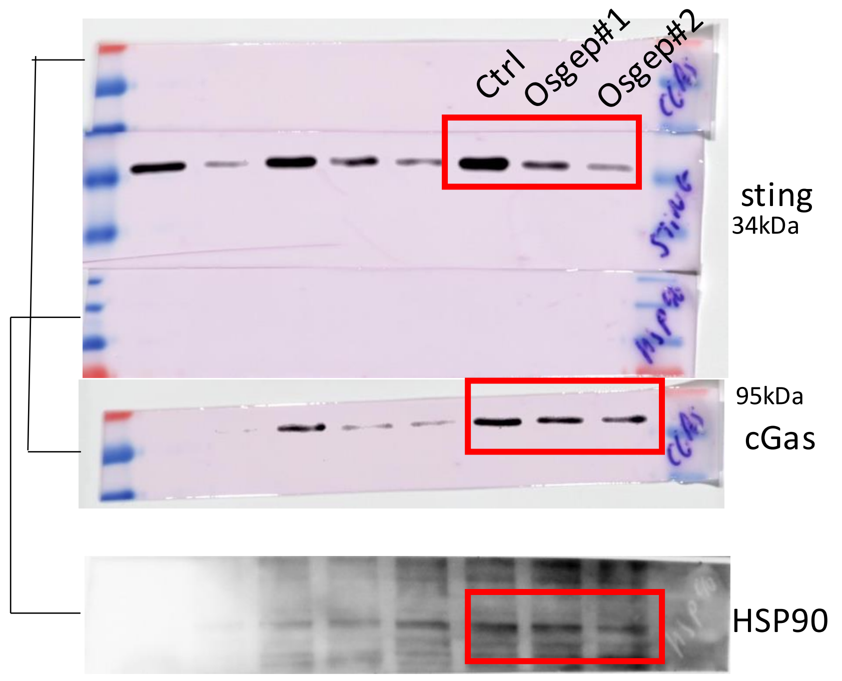

M1014 PANEL

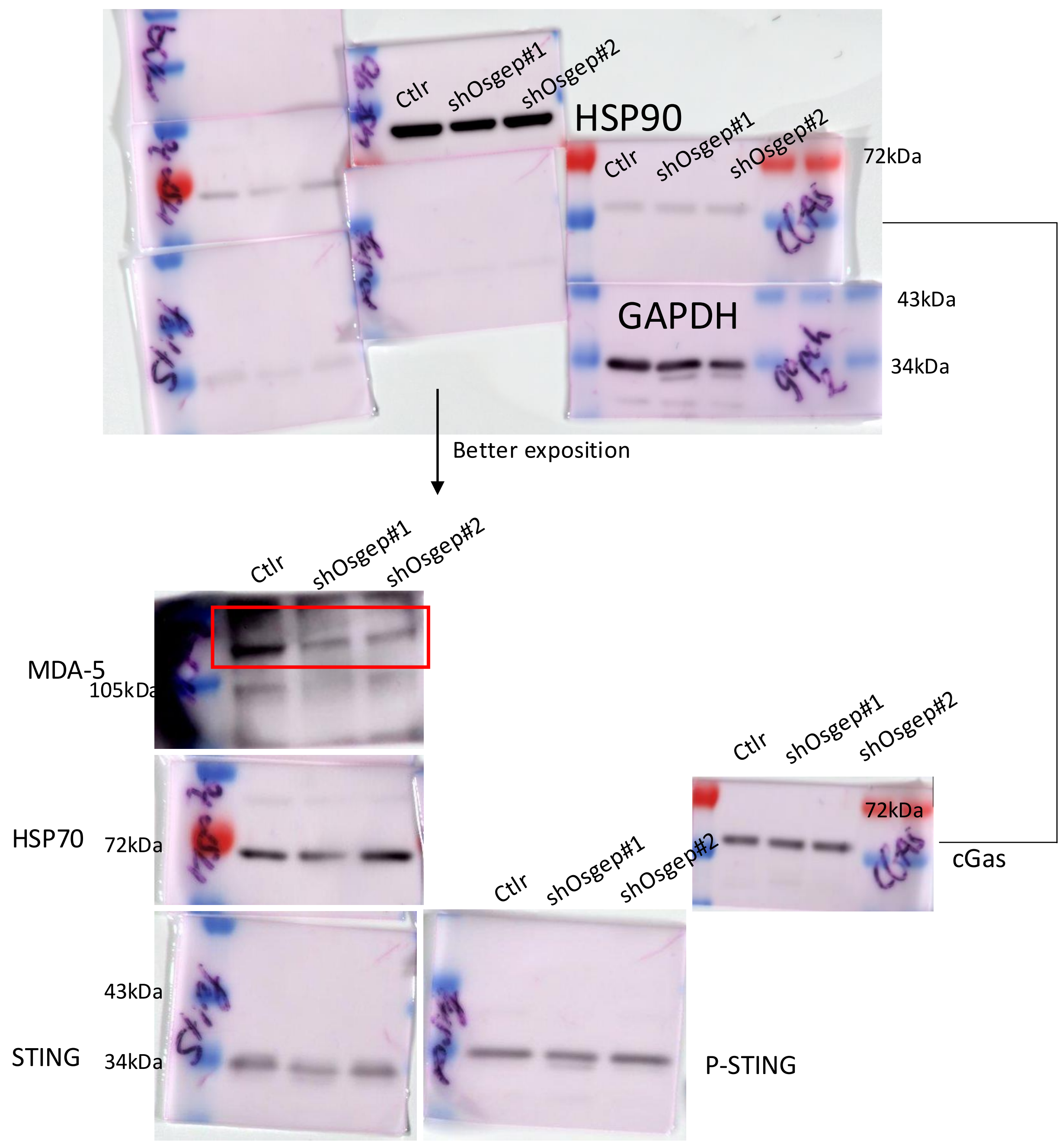

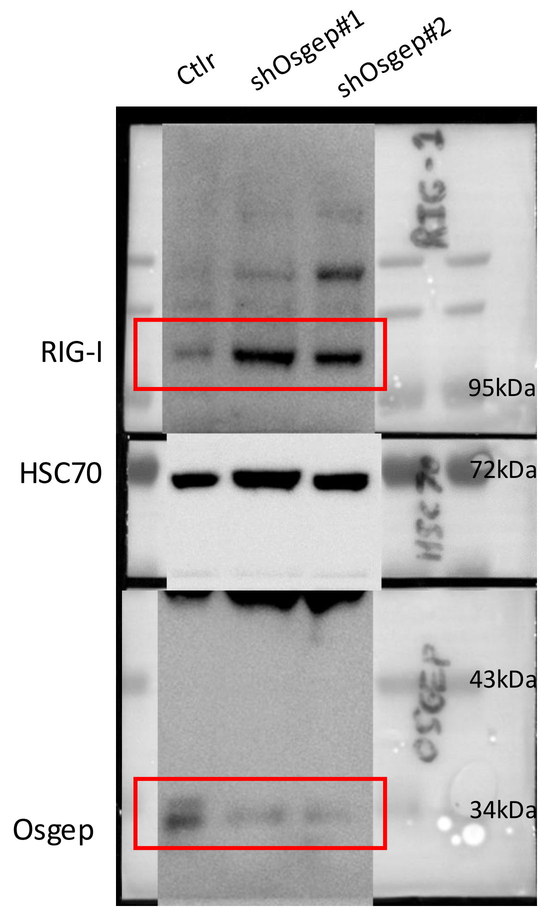

Gel from Western Blot FIGURE 3F

B16 PANEL

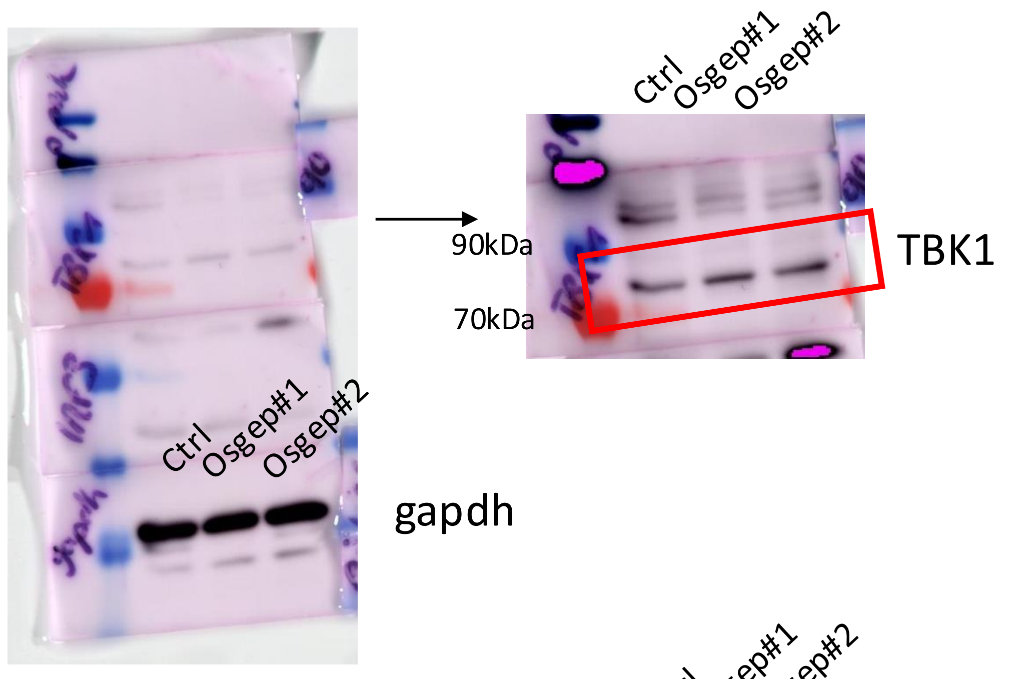

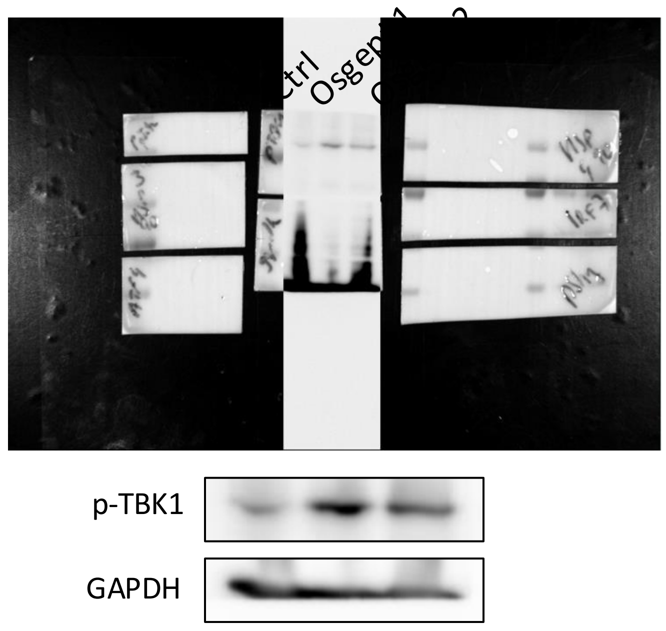

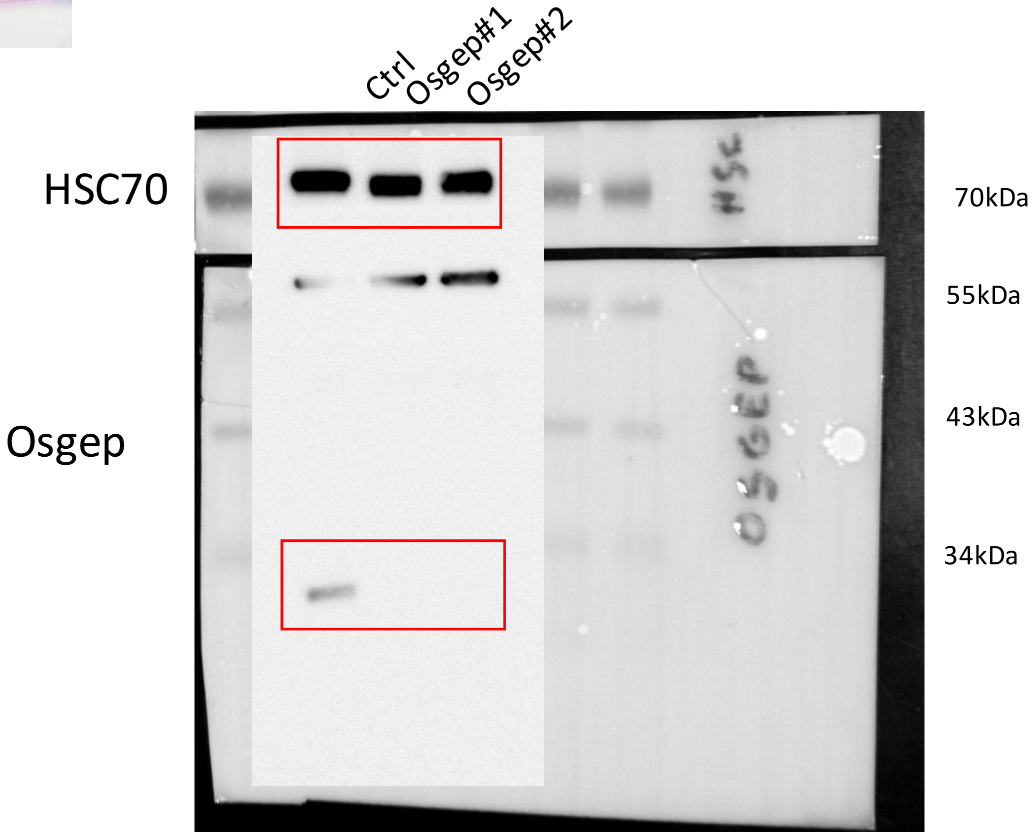

M1014 PANEL

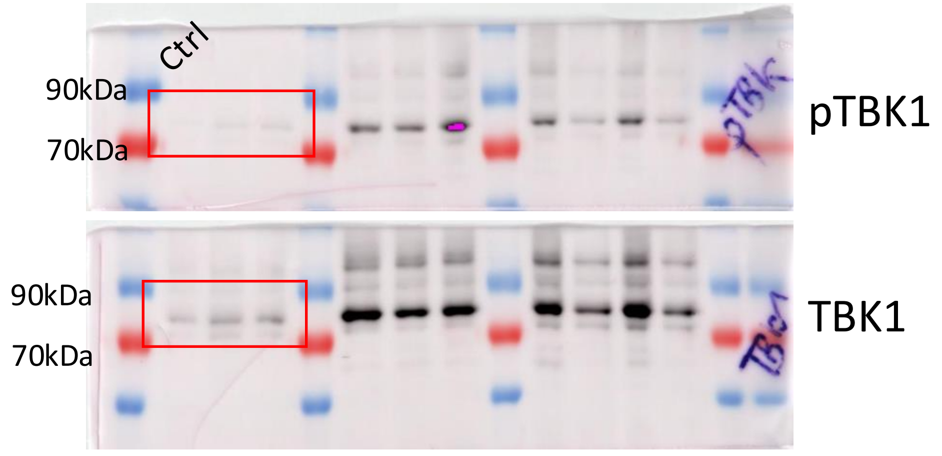

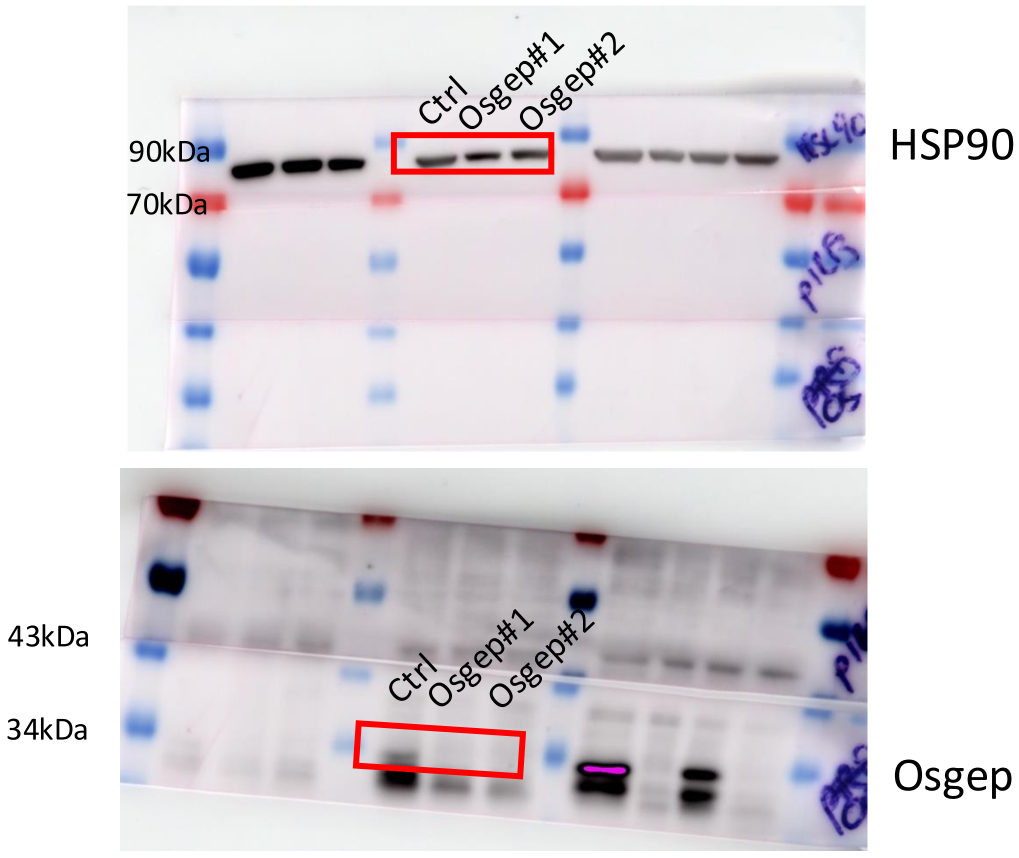

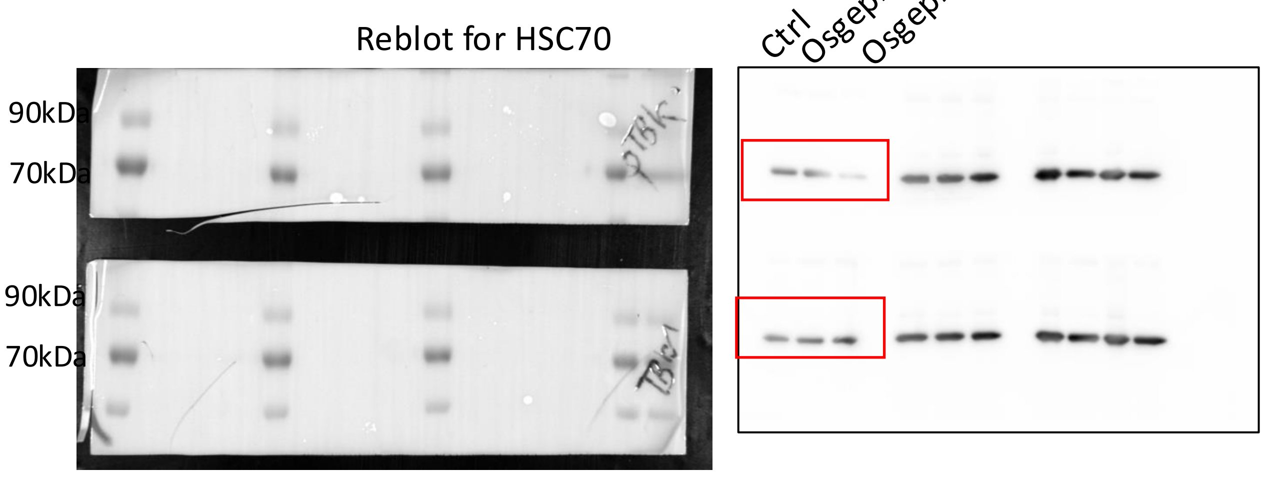

Gel from Western Blot FIGURE 5A

b16

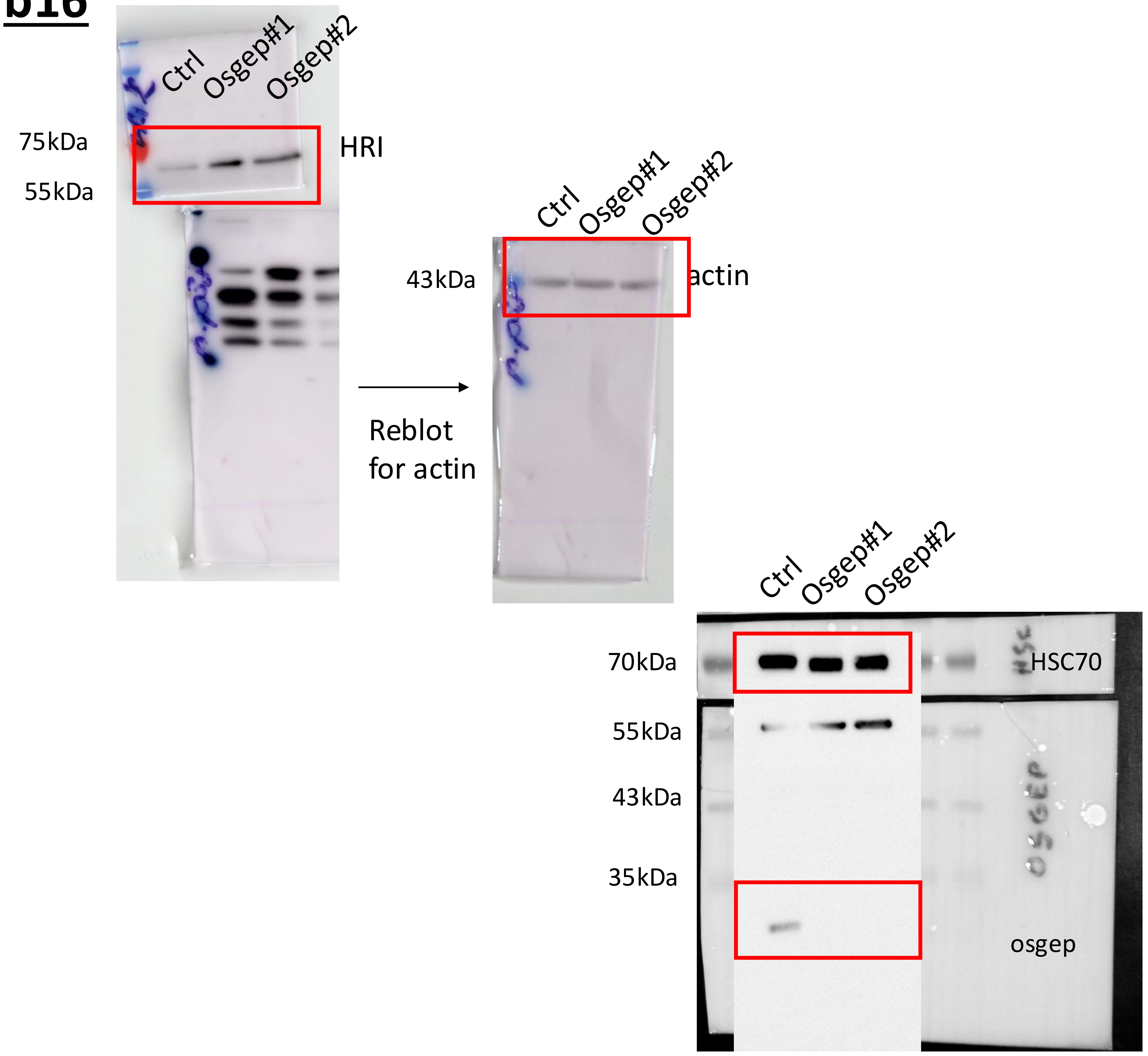

M1014

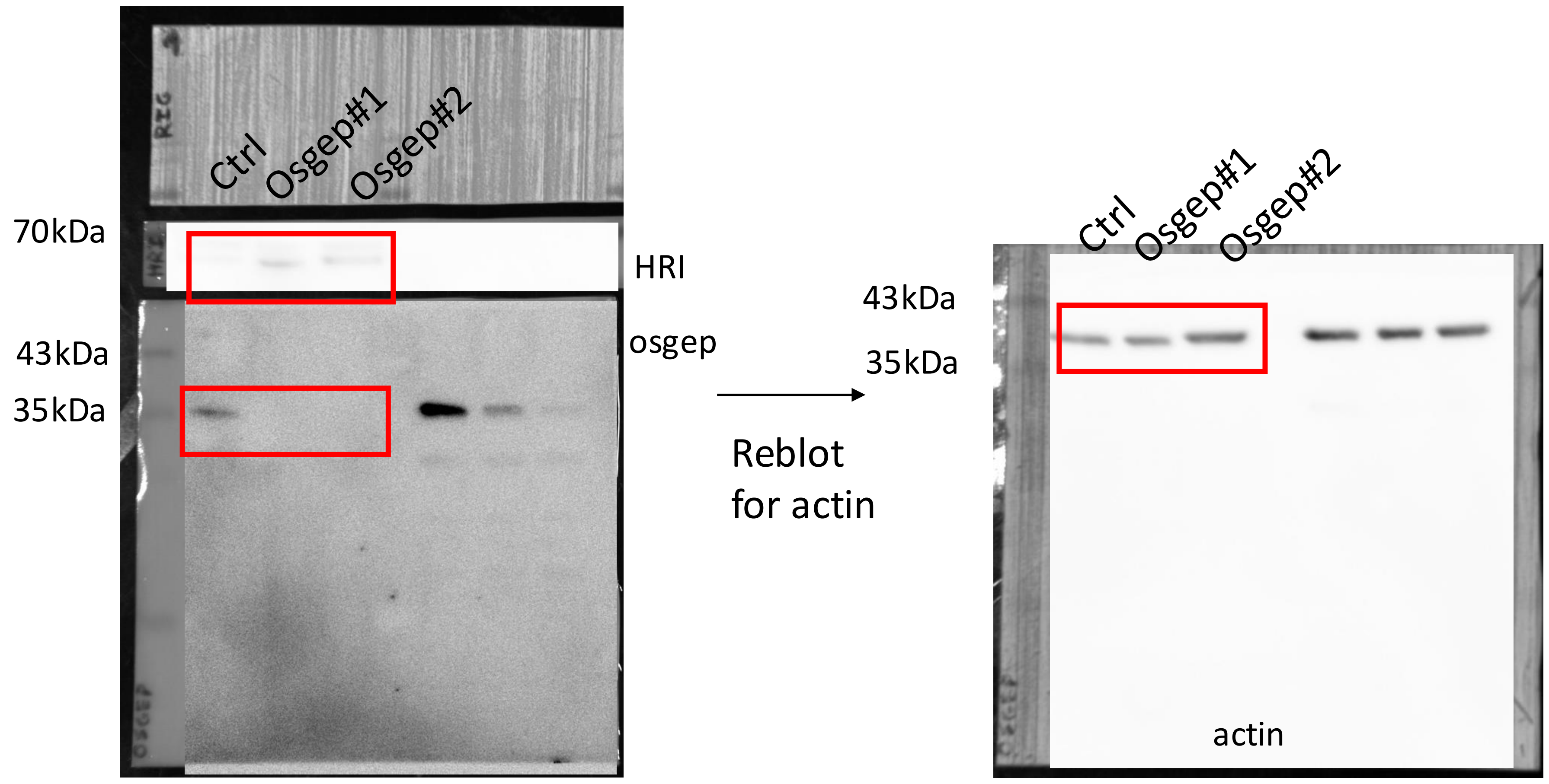

Gel from Western Blot FIGURE 5B

B16 PANEL

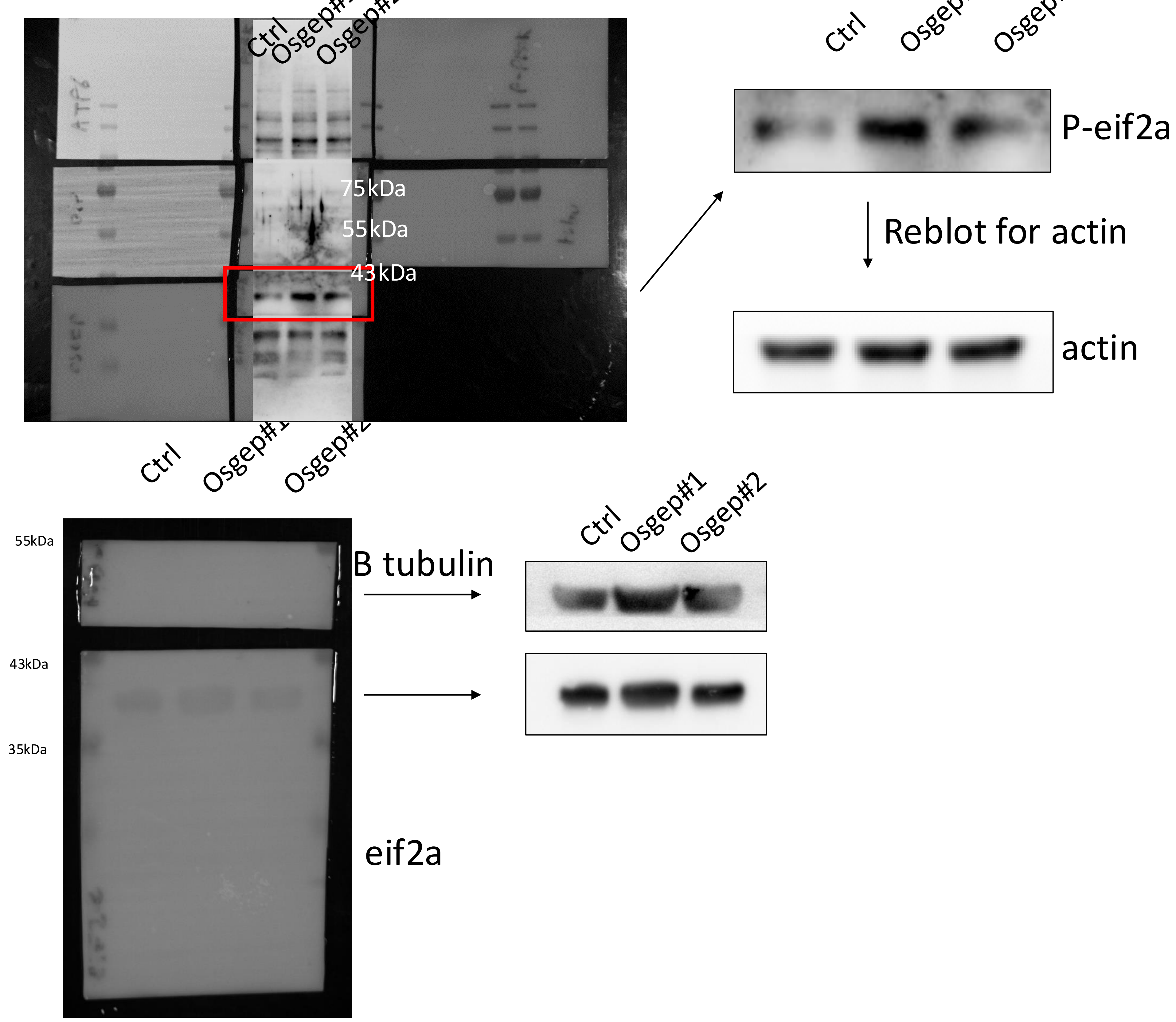

M1014 PANEL

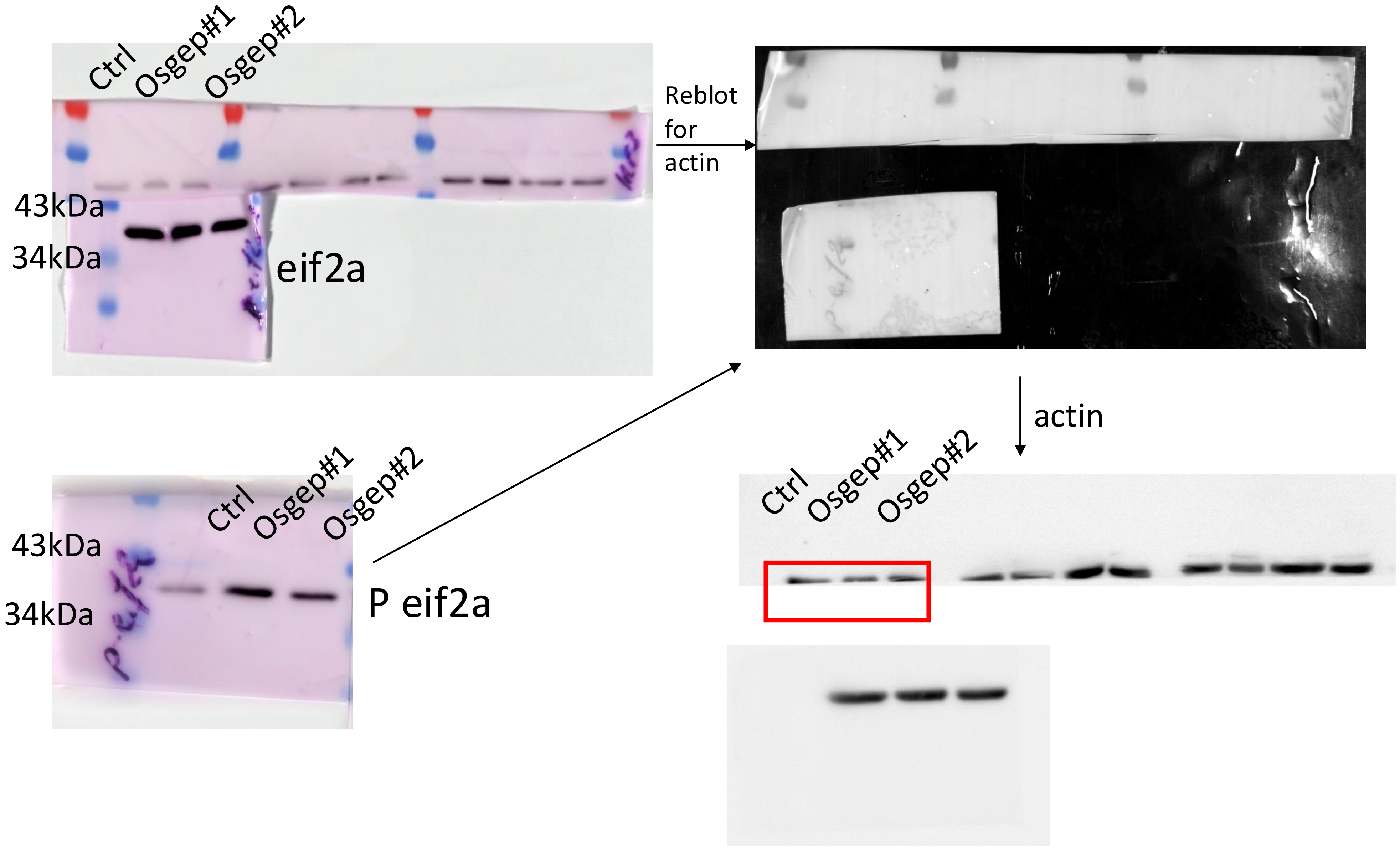

Gel from Western Blot FIGURE 5H

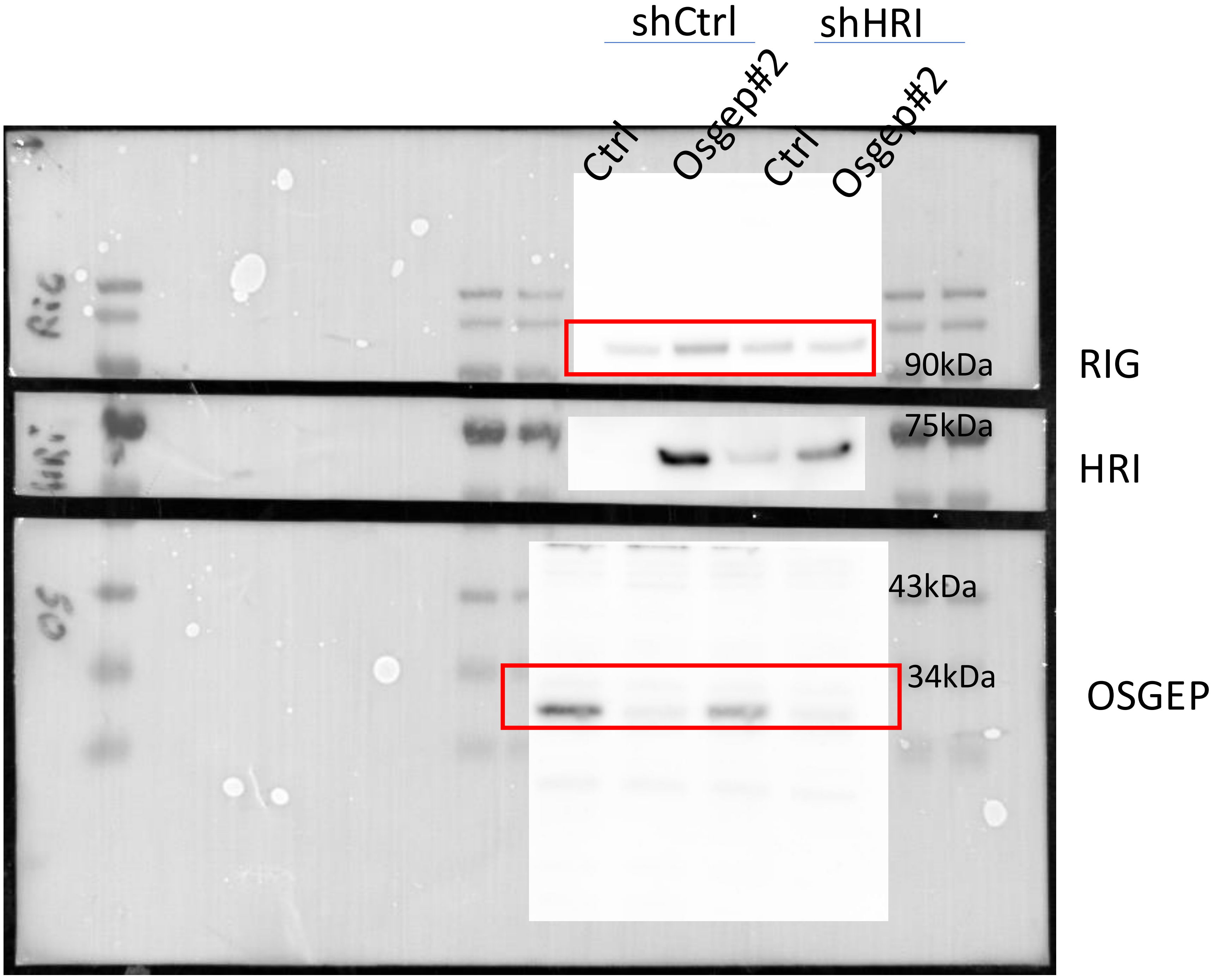

Reblot for actin

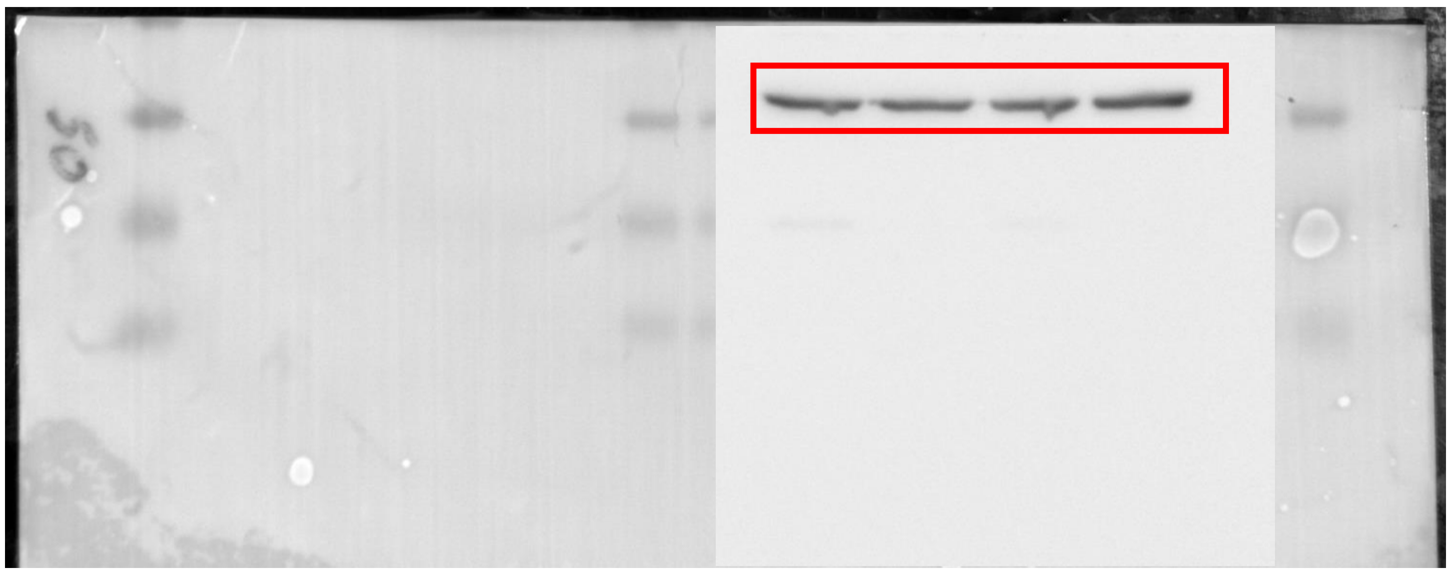

Gel from Western Blot FIGURE 6A

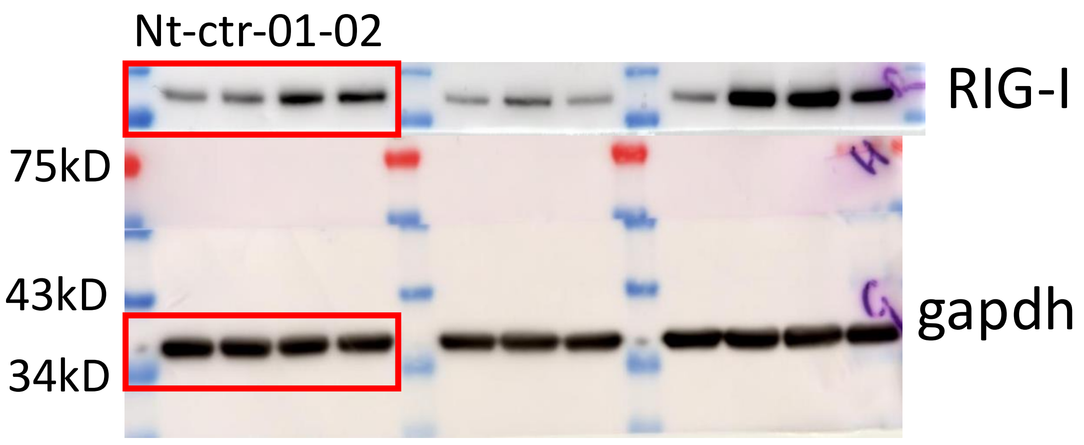

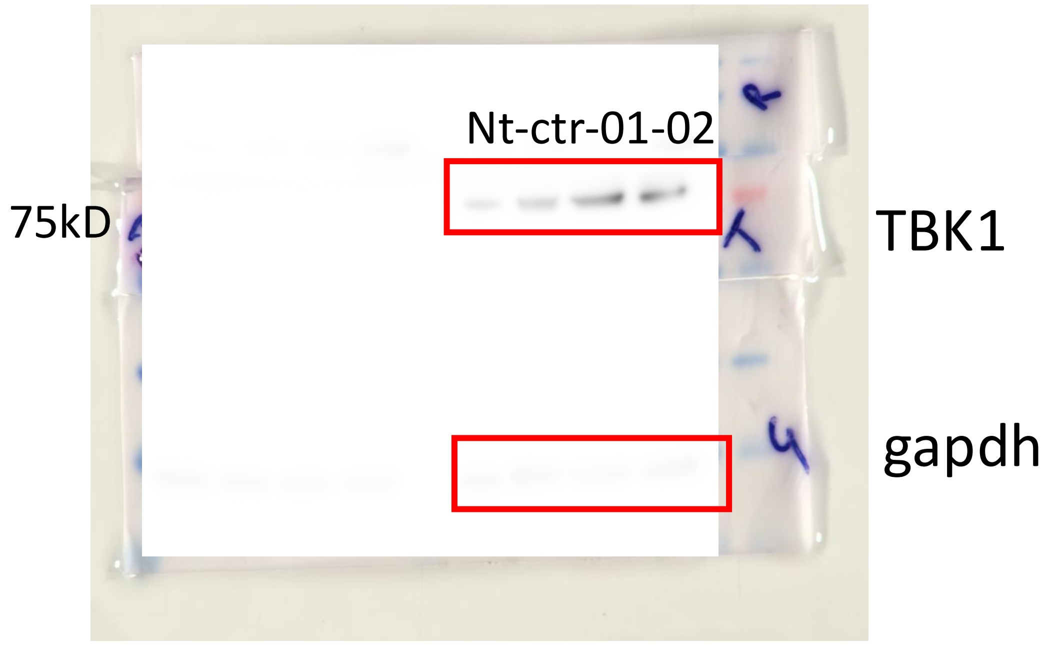

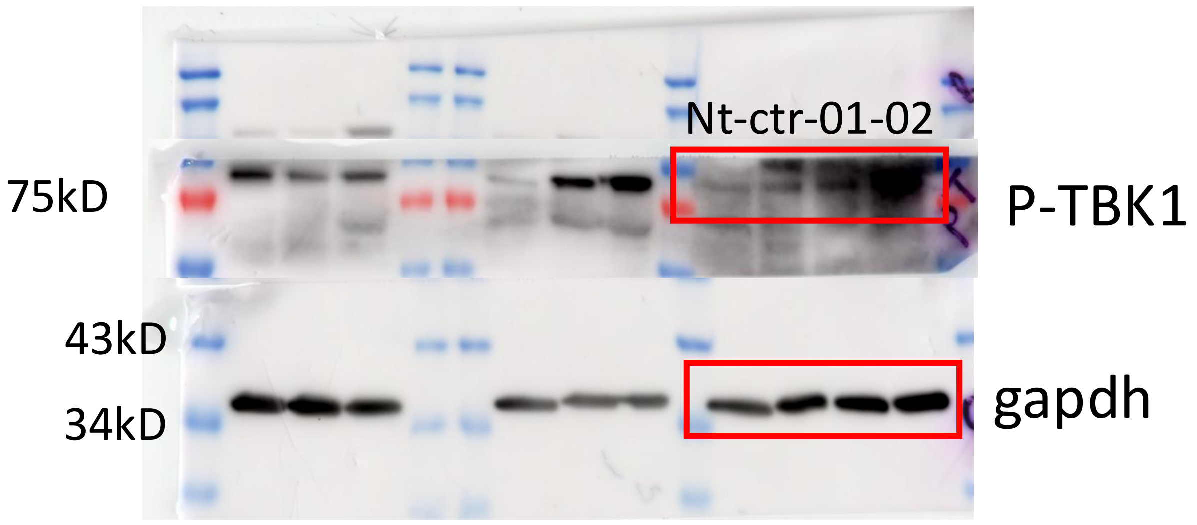

Gel from Western Blot FIGURE 6E

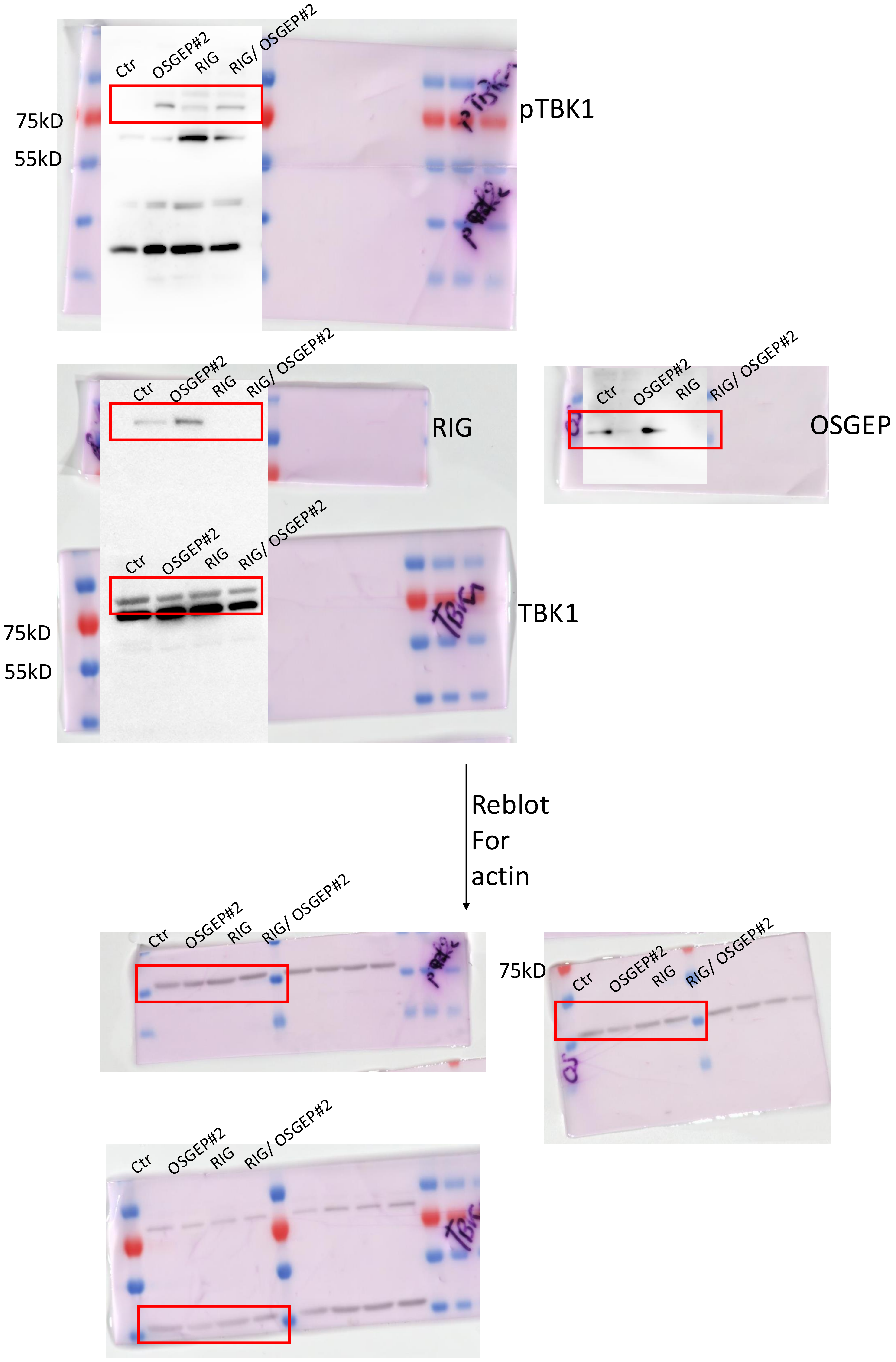

Supplement: Supplementary file 12 — Source data [file 41467_2026_69964_MOESM12_ESM.zip › Source data /Source data file_uncropped western blots.pdf]
